# Meta-analysis of tumor- and T cell-intrinsic mechanisms of sensitization to checkpoint inhibition

**DOI:** 10.1016/j.cell.2021.01.002

**Published:** 2021-02-04

**Authors:** Kevin Litchfield, James L. Reading, Clare Puttick, Krupa Thakkar, Chris Abbosh, Robert Bentham, Thomas B.K. Watkins, Rachel Rosenthal, Dhruva Biswas, Andrew Rowan, Emilia Lim, Maise Al Bakir, Virginia Turati, José Afonso Guerra-Assunção, Lucia Conde, Andrew J.S. Furness, Sunil Kumar Saini, Sine R. Hadrup, Javier Herrero, Se-Hoon Lee, Peter Van Loo, Tariq Enver, James Larkin, Matthew D. Hellmann, Samra Turajlic, Sergio A. Quezada, Nicholas McGranahan, Charles Swanton

**Affiliations:** 1 Cancer Evolution and Genome Instability Laboratory, The Francis Crick Institute, 1 Midland Road, London NW1 1AT, UK; 2 Cancer Immunology Unit, Research Department of Hematology, University College London Cancer Institute, Paul O’Gorman Building, 72 Huntley Street, London WC1E 6BT, UK; 3 Cancer Research UK Lung Cancer Centre of Excellence, University College London Cancer Institute, Paul O’Gorman Building, 72 Huntley Street, London WC1E 6BT, UK; 4 Stem Cell Group, Cancer Institute, University College London, London WC1E 6DD, UK; 5 Bill Lyons Informatics Centre, University College London Cancer Institute, Paul O’Gorman Building, 72 Huntley Street, London WC1E 6BT, UK; 6 Renal and Skin Units, The Royal Marsden NHS Foundation Trust, London SW3 6JJ, UK; 7 Department of Health Technology, Technical University of Denmark, Copenhagen, Denmark; 8 Department of Health Sciences and Technology, Samsung Advanced Institute of Health Sciences and Technology, Sungkyunkwan University, Seoul, South Korea; 9 Division of Hematology-Oncology, Department of Medicine, Samsung Medical Center, Sungkyunkwan University School of Medicine, Seoul, South Korea; 10 Cancer Genomics Laboratory, The Francis Crick Institute, 1 Midland Road, London NW1 1AT, UK; 11 Thoracic Oncology Service, Division of Solid Tumor Oncology, Department of Medicine, Memorial Sloan Kettering Cancer Center, Weill Cornell Medical College, and Parker Center for Cancer Immunotherapy, 885 2nd Avenue, New York, NY 10017, USA; 12 Cancer Dynamics Laboratory, The Francis Crick Institute, 1 Midland Road, London NW1 1AT, UK

**Keywords:** neoantigen, mutation, immunogenicity, checkpoint inhibitors, immunotherapy, biomarkers, CXCL9, clonal TMB, meta-analysis

## Abstract

Checkpoint inhibitors (CPIs) augment adaptive immunity. Systematic pan-tumor analyses may reveal the relative importance of tumor-cell-intrinsic and microenvironmental features underpinning CPI sensitization. Here, we collated whole-exome and transcriptomic data for >1,000 CPI-treated patients across seven tumor types, utilizing standardized bioinformatics workflows and clinical outcome criteria to validate multivariable predictors of CPI sensitization. Clonal tumor mutation burden (TMB) was the strongest predictor of CPI response, followed by total TMB and *CXCL9* expression. Subclonal TMB, somatic copy alteration burden, and histocompatibility leukocyte antigen (HLA) evolutionary divergence failed to attain pan-cancer significance. Dinucleotide variants were identified as a source of immunogenic epitopes associated with radical amino acid substitutions and enhanced peptide hydrophobicity/immunogenicity. Copy-number analysis revealed two additional determinants of CPI outcome supported by prior functional evidence: 9q34 (*TRAF2*) loss associated with response and *CCND1* amplification associated with resistance. Finally, single-cell RNA sequencing (RNA-seq) of clonal neoantigen-reactive CD8 tumor-infiltrating lymphocytes (TILs), combined with bulk RNA-seq analysis of CPI-responding tumors, identified *CCR5* and *CXCL13* as T-cell-intrinsic markers of CPI sensitivity.

## Highlights

• Large-scale meta-analysis of >1,000 CPI-treated cases with exome/transcriptome data
• Clonal TMB and *CXCL9/CXCL13* expression are the strongest predictors of CPI response
• A multivariable predictor of CPI response significantly outperforms TMB
• 9q34 loss and *CCND1* amplification are additional determinants of CPI response

## Graphical Abstract

**Figure:**
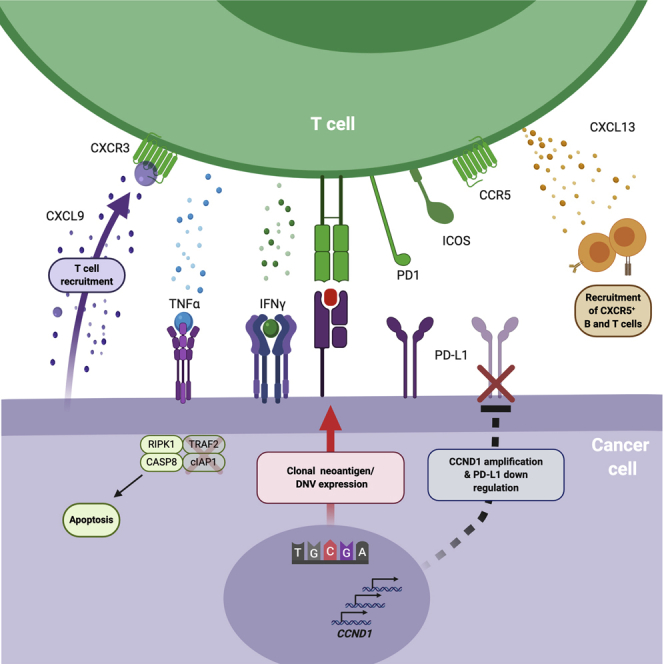

## Teaser

A whole-exome and transcriptome meta-analysis of over 1,000 patients treated with immune checkpoint blockade across seven tumor types highlights the potential of multivariable prediction models that consider both tumor- and T-cell-intrinsic mechanisms of response.

## Introduction

**Figure 1:**
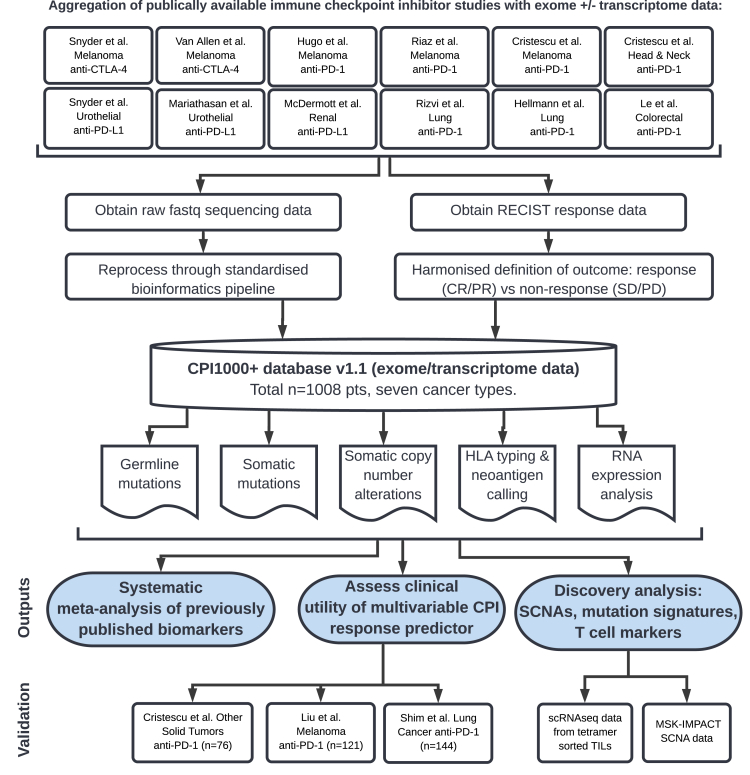
Design of the meta-analysis study Input studies to the meta-analysis (Figure 2) results (top) and validation cohorts for the multivariable predictive modeling (Figure 3) (bottom).

**Figure S1:**
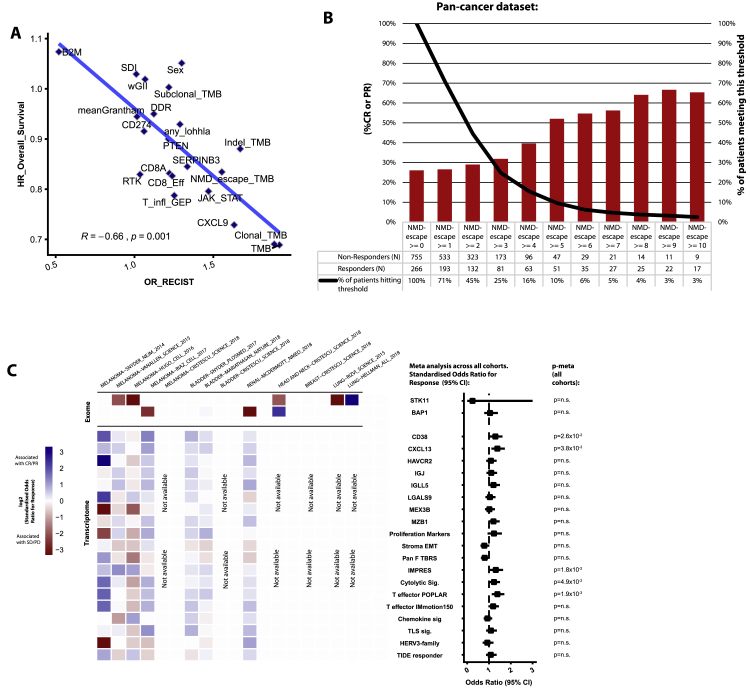
Supplementary meta-analysis data, related to Figure 2 Panel A shows the correlation in biomarker effect sizes for radiological response and overall survival clinical endpoints (Spearman's correlation). Panel B shows response rate by number of NMD-escape mutations for all available samples. Panel C shows results from previously published histology specific biomarkers, or metrics that could not be calculated in > 75% of the cohort samples.

To date, multiple biomarkers have been associated with immune checkpoint inhibitor (CPI) response, which can be broadly grouped into four categories: (1) sources of antigen that elicit T cell responses, (2) mechanisms of immune evasion that drive resistance, (3) host factors, and (4) markers of immune infiltration. Despite these promising insights, large-scale studies of CPI response in patients with in-depth whole-exome and transcriptome data have been lacking. Furthermore, given that CPIs activate the immune system rather than target cancer-cell-intrinsic pathways, we hypothesized that a systematic pan-tumor analysis could help elucidate the critical features underpinning CPI response and enable appropriately powered biomarker discovery. Accordingly, we collated raw exome/transcriptome data across multiple studies and cancer types, totaling n = 1,008 CPI-treated patients (termed the “CPI1000+ cohort”; Figure 1) from 12 individual cohorts (see STAR methods), and reprocessed these data through a uniform bioinformatics pipeline to maximize comparability across cohorts. Furthermore, we harmonized radiological clinical response definitions across the 12 studies to ensure strict consistency in outcome measurement (“responder” is defined as a RECIST-criteria-based radiological response with complete response [CR] or partial response [PR], and “nonresponder” is defined as stable disease [SD] or progressive disease [PD]). We note this is a conservative definition of response, and patients with SD and extended survival can be considered as experiencing clinical benefit from treatment; however, the “CR/PR versus SD/PD” definition allows the clearest response interpretation and is consistent with the most recent literature (Cristescu et al., 2018; Mariathasan et al., 2018). Furthermore, in a subset of patients with both radiological response and overall survival data, we found a strong relationship in biomarker effect sizes for response and survival (Figure S1A; p = 0.001). The CPI1000+ cohort comprises data from seven tumor types (metastatic urothelial cancer [n = 387], malignant melanoma [n = 353], head and neck cancer [n = 107], non-small cell lung cancer [n = 76], renal cell carcinoma [RCC] [n = 51], colorectal cancer [n = 20], and breast cancer [n = 14]), treated with three classes of CPIs (anti-CTLA-4 [n = 155], anti-PD-1 [n = 432], and anti-PD-L1 [n = 421]) (Table S1). Samples predominantly represented baseline pretreatment specimens, treated with single agent CPI, with a small number of exceptions (n = 55, 5.5%) in which the patient had either undergone prior lines of anti-CTLA-4 treatment or the biopsy was taken on treatment (see Table S1). As a validation cohort, we obtained processed copy-number segment and overall survival data from n = 1,600 cases from CPI-treated patients profiled using the MSK-IMPACT panel (Consortium, 2017; Samstein et al., 2019) (referred to hereafter as the MSK1600 cohort; RECIST response outcome data were not available for this cohort).

## Results

### Benchmarking of previously reported biomarkers of CPI response

**Figure 2:**
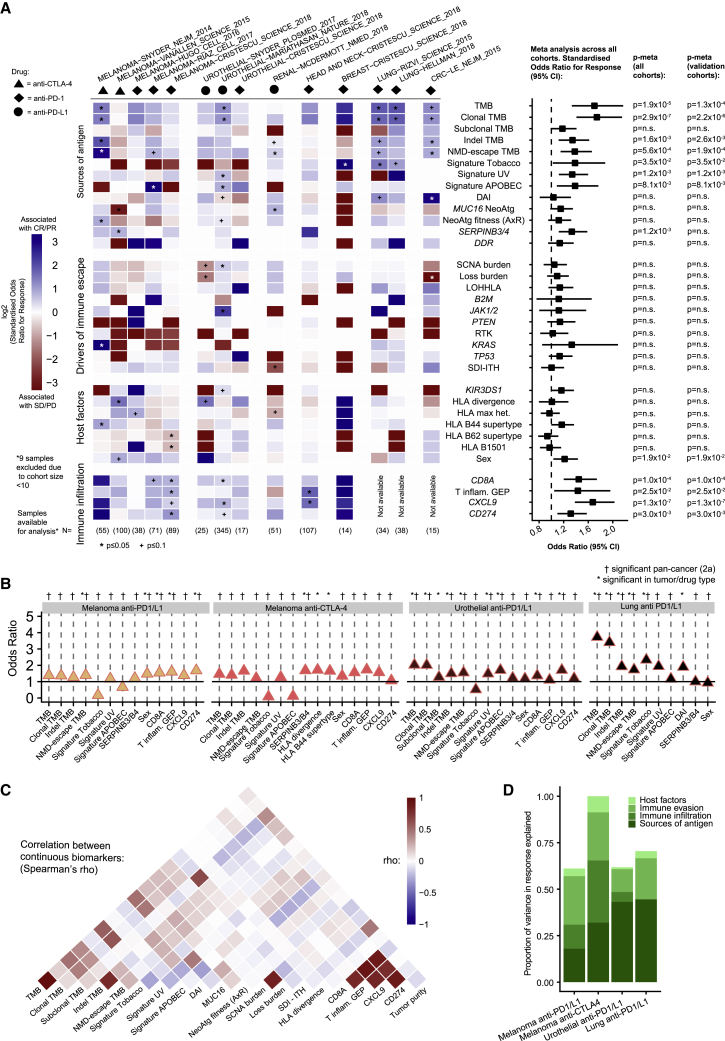
The biomarker landscape of CPI response (A) Previously published biomarkers are shown as rows and individual cohorts within the CPI1000+ cohort as columns. The heatmap indicates the effect size of each biomarker in each cohort, measured as the log2 odds ratio (OR) for response “CR/PR” versus no response “SD/PD/NE” derived from logistic regression. Blue denotes association with response, red association with no response. Drug class and cohort sizes are annotated, and the right-hand forest plot shows the overall effect size and significance of each biomarker in meta-analysis across all studies, based on effect sizes and standard errors from each individual cohort. p values are shown from meta-analysis (random effects, on account of the different tumor types), with the first set of p-values including all samples (p-meta all cohorts) and last set (p-meta validation cohorts) including validation cohorts only (i.e., when a biomarker was originally discovered in a cohort, this cohort was excluded from the meta-analysis). For clarity of plotting, outlier OR values were capped between OR = 0.1 and OR = 10 (all outlier values were nonsignificant results skewed by rare event counts, and raw (uncapped) values were still used in the meta-analysis). (B) The CPI1000+ cohort broken into cancer/drug subgroups for combinations with two or more independent cohorts. OR effect sizes are shown on the y axis, and biomarkers that are either significant in the pan-cancer 2A analysis or within an individual subgroup are shown. Colors are arbitrary and are used only to distinguish the groups. (C) Correlation between biomarkers that are measured on a continuous scale. (D) Proportion of variance explained for each category of biomarker, for each study, calculated using logistic regression pseudo-*R*^*2*^.

Samples were processed from raw sequencing reads, and standardized processing/quality control procedures were executed as described in STAR methods. We began the analysis by benchmarking previously published predictors of CPI response using a literature search to systematically identify relevant biomarkers. We reviewed 723 articles that matched the search terms (see STAR methods), yielding a panel of 55 unique biomarkers (methods). To allow biomarkers with varying measurement scales (e.g., mutation counts versus gene expression values) to be compared equivalently based on effect size rather than p value (Wasserstein et al., 2019), all biomarker values were converted to standard *Z* scores. We note *Z* score conversion has been similarly applied in other large-scale tumor mutation burden (TMB) projects (Vokes et al., 2019), and as a control all analyses were repeated without *Z* score conversion, with the top-ranked biomarkers found to be the same (data not shown). Finally, to avoid data pooling (Bravata and Olkin, 2001), each biomarker in each study was analyzed individually, and then the effect sizes/standard errors were combined through meta-analysis (Figure 2A).

The biomarker with strongest effect size across all 12 studies in the CPI1000+ cohort was clonal TMB (i.e., the number of nonsynonymous mutations estimated to be present in every cancer cell) (odds ratio [OR] for “CR/PR” versus “SD/PD” = 1.74; 95% confidence interval [CI], [1.41–2.15], p = 2.9 × 10^−7^), closely followed by total TMB (OR = 1.70 [1.33–2.17], p = 1.9 × 10^−5^). Subclonal mutation burden (subclonal TMB) was not significantly associated with CPI response (OR = 1.18 [0.99–1.41], p = 0.07), indicating that the dominant biomarker associated with CPI response is clonal mutational burden specifically. We note, however, that the single-region nature of this study, combined with tumor purity and modest sequencing depths, means we are underpowered to detect the full subclonal mutation burden of each tumor. Within the sources of antigen category, other biomarkers such as frameshift insertion/deletion burden (indel TMB) (OR = 1.34 [1.12–1.62], p = 1.6 × 10^−3^), nonsense-mediated decay (NMD) escaping (NMD-escape) fs-indel burden (OR = 1.38 [1.15–1.66], p = 5.6 × 10^−4^), proportion of mutations fitting tobacco (OR = 1.39 [1.02–1.88], p = 3.5 × 10^−2^), UV (OR = 1.34 [1.12–1.60], p = 1.2 × 10^−3^), and APOBEC (OR = 1.39 [1.09–1.76], p = 8.1 × 10^−3^) mutation signatures, as well as *SERPINB3* mutations (OR = 1.33 [1.12–1.59], p = 1.2 × 10^−3^), were all significantly associated with CPI response. Regarding nonsense mediated decay, we note CPI response rates are particularly elevated (∼50%–70% CR/PR) in patients with ≥5 fs-indel NMD-escaping mutations (Figure S1B). Within the sources of antigen category, DNA damage response pathway mutations were not associated with CPI response (OR = 1.14 [0.95–1.36, p = 0.17]), nor was the differential agretopicity index (OR = 1.03 [0.81–1.32, p = 0.79]), *MUC16* neoantigen count (OR = 1.15 [0.98–1.35, p = 0.08]), or AxR neoantigen fitness model (OR = 1.12 [0.95–1.32, p = 0.18]). With regard to drivers of immune escape, we observed no significant association between the level of somatic copy-number alteration (SCNA), measured using the weighted genome instability index (wGII) (Endesfelder et al., 2014), and CPI response (OR = 1.05 [0.87–1.25], p = 0.62), or copy-number loss burden (OR = 1.09 [0.93–1.28], p = 0.27). *B2M*, *PTEN*, *JAK1/JAK2*, *KRAS*, *TP53*, and receptor tyrosine kinase (RTK) mutations did not reach overall significance, despite showing strong effect sizes in some individual cohorts (see Figure 2A), nor did the ITH Shannon diversity index (Wolf et al., 2019). Intriguingly, loss of heterozygosity (LOH) at the human leukocyte antigen (HLA) locus (LOHHLA) (McGranahan et al., 2017) had a non-significant OR in the direction of improved chances of CPI response (OR = 1.14, [0.95–1.36, p = 0.16), the opposite of what may be expected, and possibly reflecting the fact that LOHHLA is found at higher frequency later in tumor evolution and is enriched in hot versus cold tumors (Rosenthal et al., 2019). As a technical validation LOHHLA analysis was repeated using: (1) the ASCAT tool (Van Loo et al., 2010) to call LOH, and (2) only calls concordant between ASCAT and LOHHLA tools. Both of these additional analyses yielded the same result (i.e., non-significant OR numerically > 1). Regarding host factors, we did not observe a significant association between the level of germline HLA-I evolutionary divergence (Chowell et al., 2019) (OR = 1.01 [0.80–1.28], p = 0.94) in the combined meta-analysis, nor for maximal HLA heterozygosity (OR = 0.97 [0.83–1.14], p = 0.70), HLA B62 supertype (OR = 0.93 [0.78–1.11], p = 0.45), HLA B1501 type (OR = 0.97 [0.81–1.16], p = 0.75) (Chowell et al., 2018), or germline variants in the *KIR3DS1* gene (OR = 1.16 [0.99–1.37], p = 0.067). HLA B44 supertype was found to be marginally nonsignificant (OR = 1.17 [1.00–1.37], p = 0.053), and sex was found to have a significant association (OR = 1.22 [1.03–1.43, p = 1.9 × 10^−2^), with male patients experiencing better response rates as previously described (Conforti et al., 2018). In the markers of immune infiltration category, we observed *CXCL9* expression (House et al., 2020) as the predictor with strongest effect size (OR = 1.67 [1.38–2.03], p = 1.3 × 10^−7^), followed by significant associations for *CD8A* expression (OR = 1.45 [1.20–1.74], p = 1.0 × 10^−4^), the T cell inflamed gene expression signature (Ayers et al., 2017) (OR = 1.43 [1.05–1.96], p = 2.5 × 10^−2^), and *CD274 (*PD-L1) expression level (OR = 1.32 [1.10–1.58], p = 3.0 × 10^−3^). CXCL9 is a critical chemokine that binds CXCR3 on T cells, enhancing recruitment of cytotoxic CD8^+^ T cells into the tumor (Gorbachev et al., 2007) and promoting the differentiation of inflammatory T helper type 1 (Th1) and Th17 CD4 T cells (Karin et al., 2016). Additional biomarkers identified in the literature review that are either histology specific or could not be measured in >75% of samples are included in Figure S1C. Of these, the following were significant: *CD38* expression (OR = 1.29 [1.03–1.61, p = 2.6 × 10^−2^), *CXCL13* expression (OR = 1.38 [1.11–1.73, p = 3.8 × 10^−3^), IMPRES (OR = 1.31 [1.05–1.65, p = 1.8 × 10^−2^), T effector signature from the POPLAR trial (OR = 1.38 [1.13–1.70, p = 1.9 × 10^−3^), and cytolytic score (OR = 1.22 [1.00–1.51, p = 4.9 × 10^−2^). Three signatures (stroma-EMT/pan-fibroblast transforming growth factor β (TGF-β)/T effector score from IMmotion150 trial), while nonsignificant, had p < 0.1 (Figure S1C).

**Figure S2:**
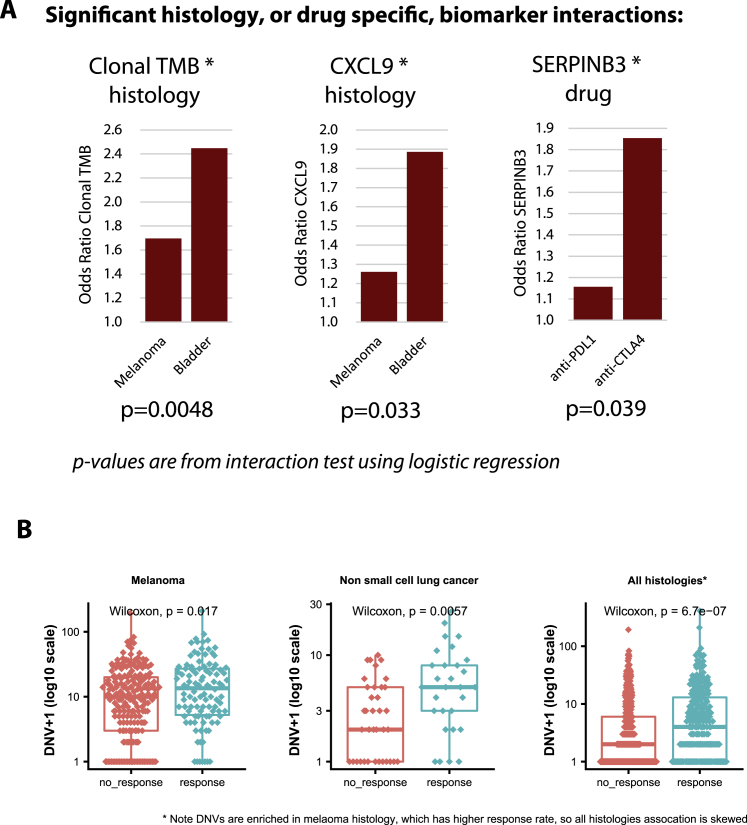
(A) shows significant histology or drug-specific biomarker interactions identified in the CPI1000+ cohort (using histology*biomarker and drug*biomarker interaction terms in logistic regression), and (B) shows dinucleotide variant associations with CPI response, related to Figures 2 and 4

We note that the lack of a statistically significant association for any of these biomarkers does not rule out an important underlying biological role for these processes in determining CPI response. Instead, these data provide insights into the universal predictors of CPI response, with evidence of predictive utility across multiple tumor types. Furthermore, for rarer mutational events, this analysis is underpowered (e.g., *B2M* mutations/deletions were found only in 1.4% of cases), meaning larger sample sizes are likely required to confirm the role of these events in influencing CPI response. We next analyzed the CPI1000+ data split by cancer/drug type, assessing four groupings where we had two or more independent cohorts available: melanoma anti-PD-1/L1, melanoma anti-CTLA-4, urothelial carcinoma anti-PD-1/L1, and non-small cell lung cancer anti-PD-1/L1. The majority of biomarkers significant in individual subgroups were the same as those attaining significance in the pan-cancer meta-analysis, with the exception of HLA B44 supertype (Chowell et al., 2018) and germline HLA-I evolutionary divergence (Chowell et al., 2019), which, while not significant overall, were significant in the melanoma anti-CTLA-4 cohorts (OR = 1.65 [1.11–2.46], p = 1.3 × 10^−2^ and OR = 1.71 [1.07–2.75], p = 2.5 × 10^−2^, respectively). This latter association is potentially consistent with the increase in T cell receptor (TCR) diversity observed in anti-CTLA-4-treated patients (Cha et al., 2014), and hence, a broader set of HLA presented peptides may facilitate improved response; however, other explanations are possible. The only other exceptions were differential agretopicity index (significant only in lung cancer anti-PD-1/L1 cohorts [OR = 1.90 (1.05–3.44), p = 3.5 × 10^−2^]) and subclonal TMB (significant only in urothelial anti-PD-1/L1 cohorts [OR = 1.28 (1.01–1.62), p = 4.2 × 10^−2^]) (Figure 2B). Other cancer/drug histology differences reflected expected patterns; for example, *CD274* (PD-L1) was significant in melanoma anti-PD-1 cohorts, but not anti-CTLA-4 (Figure 2B). To formally test for histology/drug-specific biomarker differences, we also conducted interaction tests and found three significant interactions (Figure S2A), the first being between histology and TMB/clonal TMB, with the predictive effect size of TMB being significantly lower in melanoma as compared to urothelial carcinoma (p = 4.8 × 10^−3^) (Figure S2A). Similarly, we also observed a significantly lower OR effect size for *CXCL9* expression in melanoma as compared to urothelial cancer (p = 3.3 × 10^−2^) (Figure S2A). Third, *SERPINB3* mutations were found to have significantly higher effect size in anti-CTLA-4 versus anti-PD-1/L1 cohorts (p = 3.9 × 10^−2^) (Figure S2A). We next assessed the level of correlation between continuous biomarkers, observing a high level of correlation between metrics within each category (e.g., mutational metrics like TMB and clonal TMB were strongly correlated with each other). Similarly, markers of immune infiltration like *CD8A* and *CXCL9* were correlated with each other (Figure 2C). However, the correlation between separate biomarker categories was generally low (e.g., sources of antigen biomarkers were largely not correlated with markers of immune infiltration), suggesting potential nonredundant utility in combining multiple markers together into a multivariable test. Finally, we quantified the total proportion of variance in CPI response that could be explained by all biomarkers measured in Figure 2A, which for most studies gave a value of ∼0.6, suggesting that up to 40% of the factors determining CPI outcome are either still to be discovered or lie outside of the exome/transcriptome (Figure 2D; values calculated using logistic regression pseudo-*R*^*2*^).

### A multivariable predictor of CPI response

**Figure 3:**
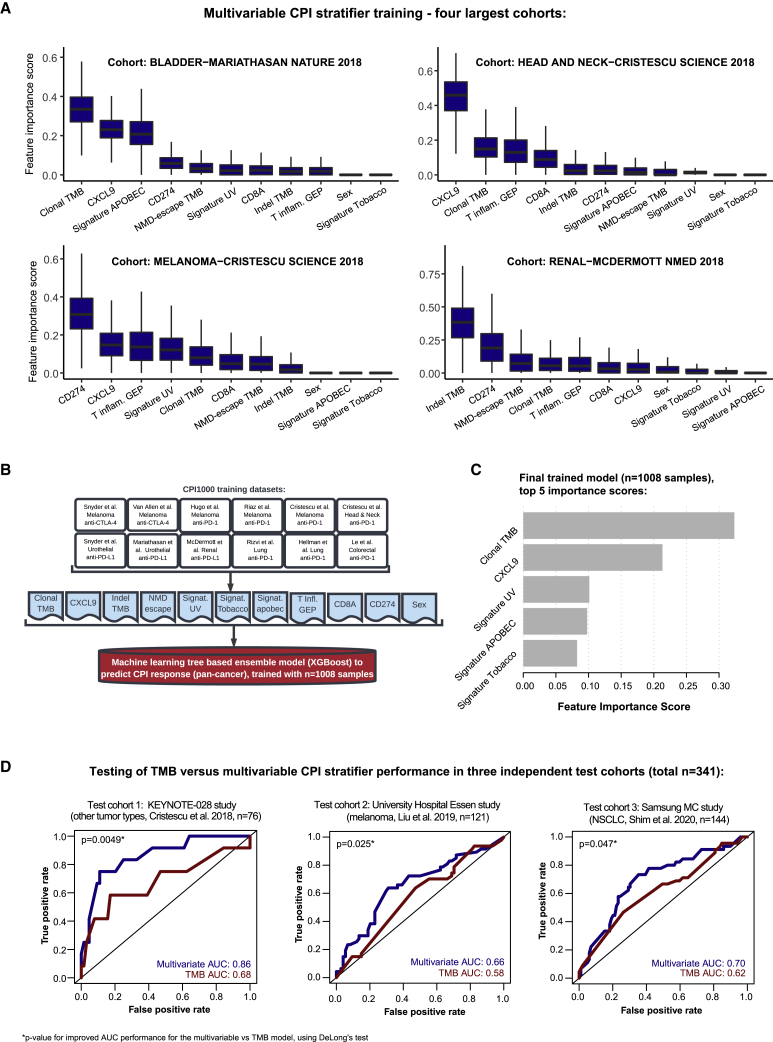
A multivariable predictor of CPI response outperforms TMB (A) Feature importance scores from XGBoost for the multivariable model, corresponding to 1,000 Monte Carlo sampling rounds. (B) Design, samples included, and features utilized in the final model training. (C) The top five feature importance scores from the final pan-cancer model. (D) ROC curves and AUC values for the multivariable predictor benchmarked to TMB, as a univariable comparator, in the three independent test cohorts (not used in any of the model training steps). p values report the significance of improved performance for the multivariable versus TMB model using DeLong’s test.

Given the complexity of the CPI biomarker landscape, we next explored if biomarkers could be combined and converted into a single score predicting the overall likelihood of CPI response with improved accuracy. For this analysis, we started by exploring the importance of predictive features in the largest training cohort of matched exome and transcriptome data for each tumor type: urothelial (n = 215; Mariathasan et al., 2018), head and neck (n = 106; Cristescu et al., 2018), melanoma (n = 89; Cristescu et al., 2018), and renal (n = 44; McDermott et al., 2018) (total across these four cohorts, n = 454). The multivariable model was trained using all biomarkers achieving overall significance in the Figure 2A meta-analysis (final column [11 total]), validation cohort results), namely clonal TMB, indel TMB, NMD-escape TMB, tobacco signature, UV signature, APOBEC signature, sex, T cell inflamed GEP signature, and gene expression values for *CD274* (PD-L1), *CD8A*, and *CXCL9*. TMB was used as a univariable benchmark comparison measure, due to it's US Food and Drug Administration (FDA) approval. We utilized a machine learning algorithm, XGBoost (see STAR methods), to construct a multivariable predictive model for each cancer type (using the 11 features described above), which demonstrated some subtle differences by cancer type (e.g., the APOBEC signature proportion was highly ranked in urothelial carcinoma and the UV signature proportion in melanoma) (Figure 3A). However, the models also displayed strong evidence of similarity. For example, clonal TMB and *CXCL9* expression were both ranked as the top two in multiple models (Figure 3A). Hence, a final combined pan-cancer model was trained using all CPI1000+ samples (n = 1,008) based on the set of 11 biomarkers listed above (Figure 3B), with feature importance scores as displayed in (Figure 3C).

In accordance with diagnostic accuracy best-practice standards, we tested the final parameterized multivariable predictor in three independent cohorts of test samples not used in the model training steps and not in the CPI1000+ cohort (test cohorts were selected based on defined criteria; see STAR methods). We calculated area under the receiver operating characteristic curve (AUC) values for the multivariable predictive model and benchmarked these to a TMB-only model for comparison purposes. Test cohort 1 was taken from KEYNOTE-028, a set of samples from Cristescu et al. (2018) (n = 76), which was selected as a test cohort due to its set of pan-cancer “other tumor type” mix of patients. The multivariable predictor attained an AUC value of 0.86, significantly better than the TMB AUC of 0.68 (p = 0.0049, DeLong’s test for comparison of AUCs; Figure 3D). Test cohort 2 was obtained from a recently published cohort from University Hospital Essen of melanoma samples (Liu et al., 2019) (n = 121), and similarly, a significantly better performance was observed for the multivariable model (AUC = 0.66) compared to the TMB AUC of 0.58 (p = 0.025, DeLong’s test; Figure 3D). Finally, test cohort 3 consisted of non-small cell lung cancer (NSCLC) samples (n = 144; obtained from Shim et al., 2020), and again, a significantly better performance was observed for the multivariable model (AUC = 0.70) compared to TMB (AUC = 0.62) (p = 0.047, DeLong’s test; Figure 3D) in the NSCLC samples. Thus, in summary, a pan-cancer multivariable model trained on n = 1,008 samples was found to significantly outperform TMB as a predictor of CPI response across three independent test cohorts, totaling ∼350 samples. Lastly, we assessed how a simpler two-parameter biomarker would perform utilizing the top biomarker from the sources of antigen and immune infiltration categories, namely clonal TMB and *CXCL9* expression. The two-parameter biomarker attained the following AUC values in each test cohort: test cohort 1 AUC = 0.79 (for reference, TMB AUC = 0.68 and full multivariable model AUC = 0.86), test cohort 2 AUC = 0.63 (for reference, TMB AUC = 0.58 and full multivariable model AUC = 0.66), and test cohort 3 AUC = 0.72 (for reference, TMB AUC = 0.62 and full multivariable model AUC = 0.70) (Table S2). Overall, while not scoring as highly as the full 11-marker model, we note that a two-marker model may have potential utility as a simplifed alternative, which is superior to TMB alone.

### Mutational processes associated with CPI response

**Figure 4:**
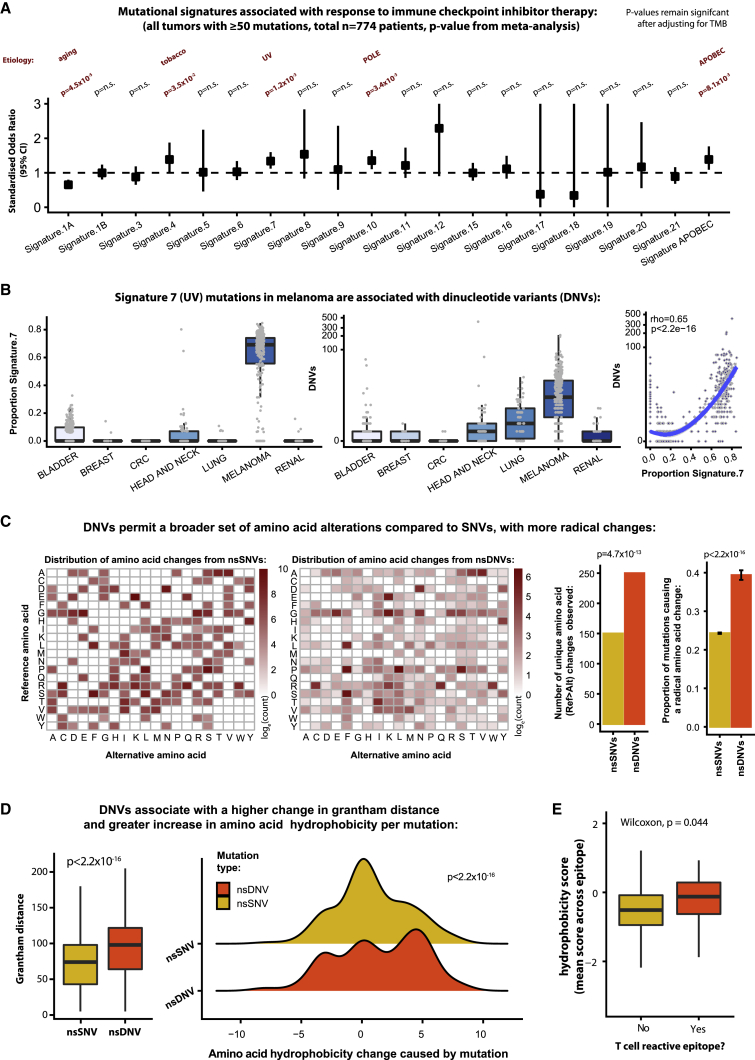
Mutational processes associated with CPI response (A) Forest plot of each mutation signature and its association with CPI response, with odds ratio values shown on the y-axis, and p-values derived from meta-analysis (hence the results are not biased by mixing histology types). (B) Proportion of signature 7 (UV) mutations (left) and the number of dinucleotide variants (DNVs) per tumor (middle), split by histology type. The panel on the right shows the correlation between signature 7 proportion and DNV count. p value and correlation coefficient are from Spearman’s rank test. (C) Grid of substitutions from the reference amino acid (rows) to the mutated amino acid (columns). The heatmap is colored from low to high, based on the simple count of each observed ref > alt change in the cohort, shown on a log10 scale. The first grid (left) shows the data for SNVs, and the second grid (middle) shows data for DNVs. The first barplot (middle) then quantifies the number of unique changes observed for SNVs and DNVs, and the second barplot shows the proportion of amino acid changes resulting in a radical amino acid change (i.e., Grantham distance ≥ 100) compared to those resulting in a conservative change (Grantham distance < 100), with p-values derived from Fisher's exact test. (D) Grantham distances for SNV and DNV changes (left boxplot), and change in hydrophobicity score in the ridge plot (right), with p-value derived from Mann-Whitney U test. (E) Hydrophobicity scores of neoantigen epitopes undergoing T cell reactivity screening, with p-value derived from Mann-Whitney U test.

Acknowledging that the current set of published biomarkers provides only a partial explanation of CPI response, we next undertook discovery analysis to search for additional predictors of response in the CPI1000+ cohort. Given the importance of TMB from the literature search, we started by assessing for evidence of mutational processes associated with treatment outcome. All samples in the CPI1000+ cohort with ≥50 somatic mutations (n = 774) were analyzed to calculate the proportion of mutations in a given sample attributable to each signature. The complete set of COSMIC mutational signatures (v2) (Alexandrov et al., 2015) was utilized. For each signature, we tested for association between the proportion of mutations fitting that signature and CPI response. To avoid any confounding bias due to different response rates across cancer types, all cohorts were analyzed individually. For example, UV signature mutations were compared within each melanoma cohort (comparing more or less sun damage within melanoma patients), and then study-level results were combined only via meta-analysis. Five out of 20 mutational processes were found to be significant: signature 1A (aging, OR = 0.65 [0.53–0.80], p = 4.5 × 10^−5^), signature 4 (tobacco, OR = 1.39 [1.02–1.88], p = 3.5 × 10^−2^), signature 7 (UV, OR = 1.34 [1.12–1.60], p = 1.2 × 10^−3^), signature 10 (POLE, OR = 1.35 [1.11–1.66], p = 3.4 × 10^−3^), and signature 2+13 (APOBEC, OR = 1.39 [1.09–1.76], p = 8.1 × 10^−3^) (Figure 4A). These associations remained significant after correction for total mutation count (i.e., TMB), suggesting that clonality and mutation quality characteristics are important. Several of these associations have also been reported by others, including tobacco (Anagnostou et al., 2020), APOBEC (Chapuy et al., 2019), and UV (Miao et al., 2018; Trucco et al., 2019). All of the signatures (except 1A, aging) were associated with a significantly improved chance of CPI response (Figure 4A). Next, we sought to identify properties of these mutational processes that may lead to more immunogenic epitopes. Interestingly, we noted a strong association between signature 4 (tobacco)/signature 7 (UV) mutations and dinucleotide variant (DNV) count. DNVs were particularly enriched in melanoma, correlating strongly with signature 7 (UV) mutation proportion (rho = 0.65, p < 2.2 × 10^−16^, Figure 4B) and significantly associated with CPI response (Figure S2B). Up to 10% of UV mutations are known to be CC > TT changes (Brash, 2015). Dinucleotide changes have two unique properties compared to single-nucleotide variants (SNVs): (1) where they straddle two codons, a double amino acid change can occur; and (2) in cases where both nucleotide changes are in the same codon, a more radical change in amino acid properties can result. While the first property is of obvious immunogenic relevance, we note only a small minority of DNVs produce a 2-amino-acid mutation (3.5% [95% CI, 3.1%–4.0%]), which in absolute number equates on average to ∼0.2 such mutations per tumor. However, the second property is of likely broader relevance, with DNVs being associated with a wider repertoire of amino acid change. Specifically, for SNV mutations, a total of only 150 unique reference to alternative amino acid change combinations were observed, whereas DNVs generated 250 different unique reference to alternative change combinations (p = 4.7 × 10^−13^, Figure 4C). Many of the amino acid changes observed in the DNV group, such as CCT codon (p = proline) change to TTT codon (F = phenylalanine), are impossible with only a single nucleotide change (Figure 4C). As such, DNVs were found to associate with a higher proportion of radical versus conservative amino acid substitutions (p < 2.2 × 10^−16^, Figure 4C), as well as a greater change in Grantham distance (p < 2.2 × 10^−16^, Figure 4D). Importantly, DNV changes were also associated with a larger increase in hydrophobicity compared to SNVs (p < 2.2 × 10^−16^, Figure 4D), a feature known to enhance peptide immunogenicity (Chowell et al., 2015). Indeed, we obtained functional data from melanoma/gastric cancer patient tumor-infiltrating lymphocyte (TIL) samples, which were screened for reactivity against neoantigen peptides (Chudley et al., 2014; Gros et al., 2016; Tran et al., 2015), and found that T cell reactive epitopes had a significantly higher hydrophobicity score compared to nonreactive epitopes (p = 0.04; Figure 4E). We note the nature of this analysis is hypothesis generating, and further functional investigation of DNVs will be of significant interest.

### Loss of 9q34.3 sensitizes tumors to CPI response

**Figure 5:**
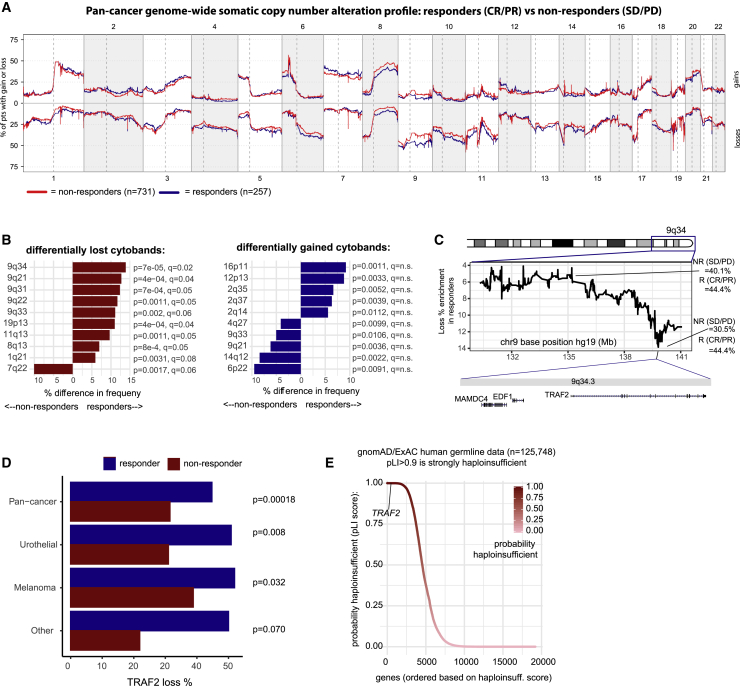
Somatic copy-number alteration (SCNA) profile of CPI responders versus nonresponders (A) Frequency of somatic copy-number gain (top) and loss (bottom) across the genome for CPI responders (“CR/PR”) versus nonresponders (“SD/PD”). (B) Cytobands with significantly different loss or gain frequencies in responders versus nonresponders,with p-value derived from Fisher's exact test, and q values from FDR correction.. (C) Fine mapping of the 9q34 locus to identify the peak of differential loss frequency between groups. (D) *TRAF2*-loss percentage frequencies for cohorts with a significant difference between responders and nonresponders, with p-value derived from Fisher's exact test. (E) Probability of haploinsufficiency (pLI) scores from the gnomAD/ExAC consortium data (n = 125,748 germline human samples).

**Figure S3:**
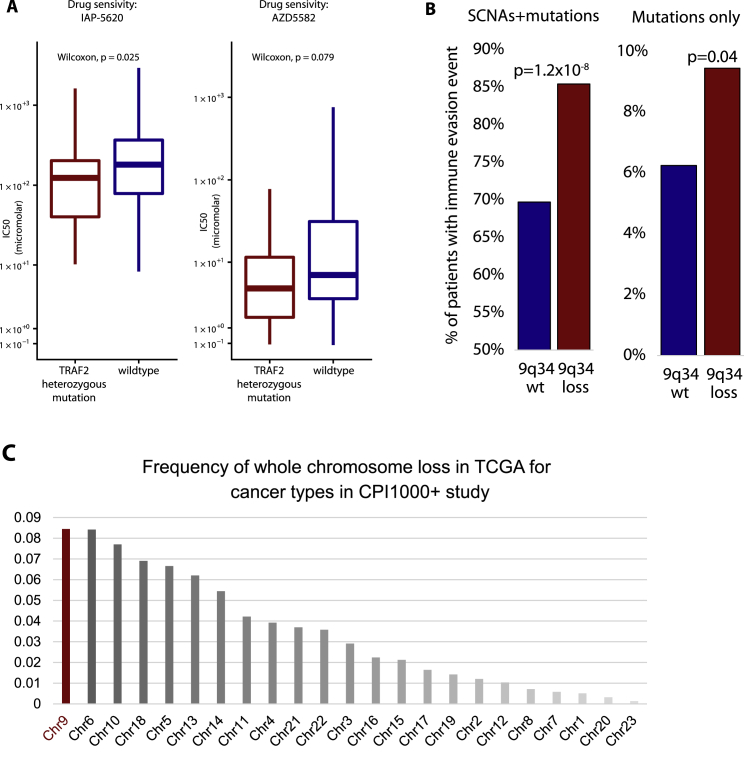
9q34 (TRAF2) analysis and immune evasion data, related to Figure 5 Panel A shows drug sensitivity screening data for two compounds, for *TRAF2* heterozygously mutated versus *TRAF2* wild-type cell lines. Panel B shows immune evasion analysis, measuring as the % of patients with an antigen presentation pathway defect between tumors with 9q34 wild-type (i.e., no loss) compared to 9q34 loss tumors. The left barplot includes either a somatic copy number loss, or a non-synonymous mutation, in an antigen presentation pathway gene. The right plot includes non-synonymous mutations only. Antigen presentation pathway genes were defined as per (Rosenthal et al., 2019), also see methods. Panel C shows the frequency of whole chromosome loss in TCGA for the set of cancer types included in the CPI1000+ study.

Next, we undertook a genome-wide somatic copy-number analysis in the CPI1000+ sample set to search for genomic loci associated with CPI response. The reasoning for this is that although the total burden of SCNAs was not found to predict response (Figure 2A), changes to specific loci may drive resistance or sensitization to therapy. The frequency of somatic copy-number gains and losses was traced across the genome for CPI responders (CR/PR) (n = 257) and nonresponders (SD/PD) (n = 731), using all samples with available QC validated exome copy-number alteration data (Figure 5A) (CPI1000+ samples, test cohort 1 and other available samples; see STAR methods), and frequency differences were compared per cytoband (Figure 5B). The most significantly differential cytoband was 9q34, which was lost in responders with a frequency of 44.4% compared to nonresponders with 30.5% (p = 6.9 × 10^−5^, q = 0.02, CPI1000+ cohort) (Figure 5B). Hence, loss of 9q34 was associated with sensitization to CPI therapy. Fine mapping of this locus revealed a sharp peak in the frequency difference at 9q34.3, directly overlapping the gene *TRAF2* (Figure 5C). *TRAF2* has been independently identified in recent functional work (Vredevoogd et al., 2019) as the top hit in a genome-wide CRISPR screen for genes, that when knocked out, sensitize tumor cells to T cell-mediated elimination. Mechanistically, *TRAF2* loss was shown to enhance CPI efficacy by lowering the tumor necrosis factor (TNF) cytotoxicity threshold and increasing T cell-mediated tumor cell apoptosis (Vredevoogd et al., 2019). *TRAF2* loss was found to be significantly enriched in responders in the overall pan-cancer cohort (p = 1.8 × 10^−4^), as well as urothelial cancer (p = 8.0 × 10^−3^), melanoma (p = 3.2 × 10^−2^), and borderline nonsignificance in the “other tumor types” cohort (p = 7.0 × 10^−2^) as individual cohorts (Figure 5D). We note the majority of 9q34 losses were found to be single-allele events (i.e., not homozygous deletions); however, supporting a potential functional impact from single-allele loss, we analyzed human germline data on n = 125,748 individuals from the gnomAD study (Karczewski et al., 2020) and found *TRAF2* to have a very high probability of being haploinsufficient (p = 0.99979, probability of haploinsufficiency [pLI] score) (Figure 5E). In addition, we obtained drug screen data from the “Genomics of Drug Sensitivity in Cancer” database (Yang et al., 2013) for two TNF pathway compounds that inhibit TRAF2 binding partners, BIRC2/BIRC3 (IAP-5620) and BIRC2 (AZD5582). Cell lines with heterozygous TRAF2 mutation (n = 32) were significantly more sensitive to IAP-5620 treatment than wild-type (n = 685) cell lines (Figure S3A; p = 2.5 × 10^−2^). Within the CPI1000+ cohort, we also observe higher rates of antigen-presentation-pathway defects, (as defined in Rosenthal et al., 2019) in 9q34 (TRAF2)-loss tumors compared to wild-type, suggesting heightened immune pressure in TRAF2-loss samples (Figure S3B; p = 1.2 × 10^−8^).

**Figure S4:**
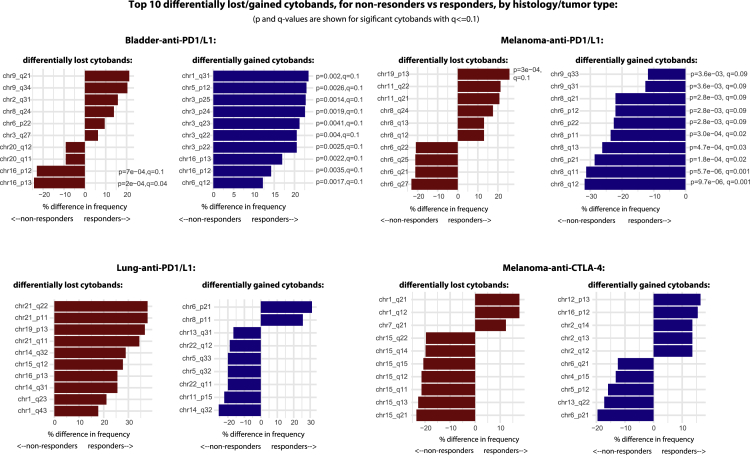
Cytobands with significantly different copy-number loss or gain frequencies in responders versus nonresponders, related to Figure 5 Analysis is split by 4 tumor/drug types.

The high frequency of 9q34 loss raises an important evolutionary question as to why tumors would be selected with a potentially disadvantageous event. Detailed inspection of the 9q34-loss events revealed that the majority of cases were in fact whole-chromosome 9 losses, and analysis of independent TCGA data for the same seven histologies considered in the CPI1000+ cohort revealed that loss of chromosome 9 is the most frequent whole-chromosome (p+q)-loss event (Figure S3C). Chromosome 9 contains a number of tumor suppressor genes, with loss of *CDKN2A* (9p21.3) in particular being under strong positive selection and associated with aggressive tumor growth in multiple tumor types (Smith and Sheltzer, 2018; Turajlic et al., 2018; Watkins et al., 2020). By contrast, loss of *TRAF2* is not documented as a cancer-driver event (e.g., not listed in the Cancer Gene Census; https://cancer.sanger.ac.uk/census), and hence, loss of this gene may be a passenger event. Following CPI treatment, the potentially deleterious impact of *TRAF2* loss on tumor cell fitness is revealed, where it has potential to enhance anti-tumor T cell activity (Vredevoogd et al., 2019). Hence, these data suggest an evolutionary model where loss of whole chromosome 9 is selected as a driver event early in tumor evolution (e.g., due to *CDKN2A*), but then later leads to collateral sensitivity (Zhao et al., 2016) to immunotherapy, possibly due to 9q34 (*TRAF2*) loss. We note chromosome 9q34 loss was also identified in a recent RCC anti-PD-1 study (Braun et al., 2020), as associated with increased immune cell infiltration. However, the histology differences between RCC and the pan-cancer cohort presented here should be noted, and hence, these findings may not be linked to a common biological cause. We acknowledge our findings here of 9q34 (*TRAF2*) loss being associated with CPI sensitization are exploratory in nature and have not been externally validated. Lastly, while primarily powered for pan-cancer copy-number analysis, we also repeated the above copy-number analysis per tumor/drug type (as per Figure 2B) and identified a number of candidate cytobands significantly associated with CPI response in individual subcohorts (q < 0.1; Figure S4).

### Focal amplification of *CCND1* associates with CPI resistance

**Figure 6:**
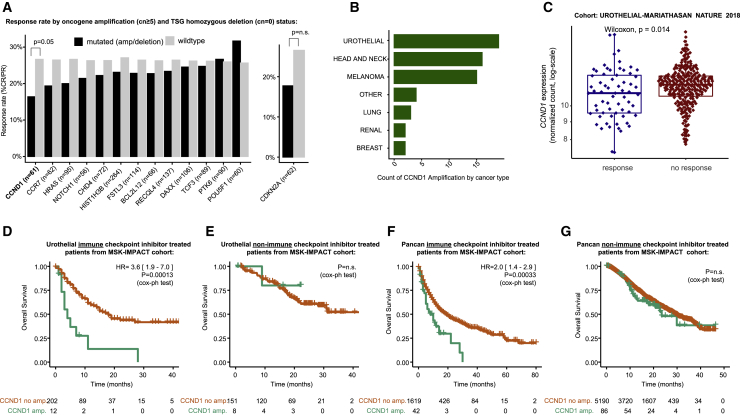
Focal amplification and deletion profile of CPI responders versus nonresponders (A) CPI response rate (% “CR/PR”) in patients with focal amplification (defined as copy number ≥ 5) or homozygous deletion (copy number = 0) compared to wild-type (nonamplified/deleted) tumors. The analysis was conducted for all oncogenes/tumor suppressor genes with greater than 5% amplification/deletion frequency, and p-values were derived from Fisher's exact test. (B) Counts of *CCND1* amplification by histology. (C) mRNA expression for *CCND1* in responders versus nonresponders from the Mariathasan et al. urothelial cancer cohort, with p-value derived from Mann-Whitney U test. (D) Overall survival analysis in MSK-IMPACT urothelial cancer CPI-treated patients for *CCND1*-amplified versus wild-type tumor groups. (E) Overall survival analysis in MSK-IMPACT urothelial cancer non-CPI-treated patients for *CCND1*-amplified versus wild-type tumor groups. (F) Overall survival analysis in MSK-IMPACT pan-cancer CPI-treated patients for *CCND1*-amplified versus wild-type tumor groups. (G) Overall survival analysis in MSK-IMPACT pan-cancer non-CPI-treated patients for *CCND1*-amplified versus wild-type tumor groups.

Next, we considered focal (<3 Mb) (Krijgsman et al., 2014) amplifications (defined as copy number ≥ 5) (Fontanilles et al., 2020) and homozygous deletions (copy number = 0) in oncogenes and tumor suppressor genes respectively, to understand if these events are associated with CPI response. The most significant association was found to be significantly lower rates of CPI response in tumors with *CCND1* amplification (response rate = 16.3%) compared to wild-type (26.6%) (p = 4.8 × 10^−2^; Figure 6A). Similarly to *TRAF2*, prior functional evidence supports a role for *CCND1* in determining CPI response (Zhang et al., 2018). Specifically, Zhang et al. (2018) demonstrated that PD-L1 protein abundance fluctuates during cell-cycle progression and that Cyclin D-CDK4 negatively regulates PD-L1 protein stability. Urothelial carcinoma had the highest number of *CCND1* amplified tumors (Figure 6B); accordingly we assessed mRNA levels in this histology type and observed significantly higher levels of *CCND1* expression in urothelial cancer nonresponders (SD/PD) versus responders (PR/CR) (p = 1.5 × 10^−2^) (Figure 6C). To validate the effect of *CCND1* amplification in an independent cohort, we conducted overall survival analysis in n = 214 urothelial cancer patients treated with CPI in the MSK1600 cohort and observed a strong effect size whereby *CCND1* amplification was associated with significantly shorter overall survival (hazard ratio [HR] = 3.6 [1.9–7.0], p = 1.3 × 10^−4^)(Figure 6D). As negative control, we observed no overall survival difference in MSK-IMPACT urothelial cancer patients not treated with CPI, controlling for the possibility that *CCND1*-amplified tumors have a generally poorer prognosis irrespective of CPI treatment (Figure 6E). Finally, we assessed the role of *CCND1* amplification in a pan-cancer context in MSK1600 and found a significant association with reduced overall survival in CPI-treated patients (HR = 2.0 [1.4–2.9], p = 3.3 × 10^−4^) (Figure 6F), but not the non-CPI-treated MSK cohort (p = n.s., which is a larger cohort with arguably greater power) (Figure 6G). The data suggest a predictive association between *CCND1* amplification and CPI resistance, rather than prognostic; however, formal treatment × genotype interaction analysis will be required to confirm this (we note the two MSK cohorts, CPI and non-CPI, had considerably different follow-up times and could not be reliably combined together for interaction analysis; we further note the sensitivity to call single-allele SCNA events was found to be reduced in the MSK panel data, which was not encountered for validation of *CCND1* amplification events which have multiple copy gains; therefore, we could not reliably address 9q34 (*TRAF2*) losses in this cohort).

### Single-cell RNA-seq identifies *CXCL13* and *CCR5*

**Figure 7:**
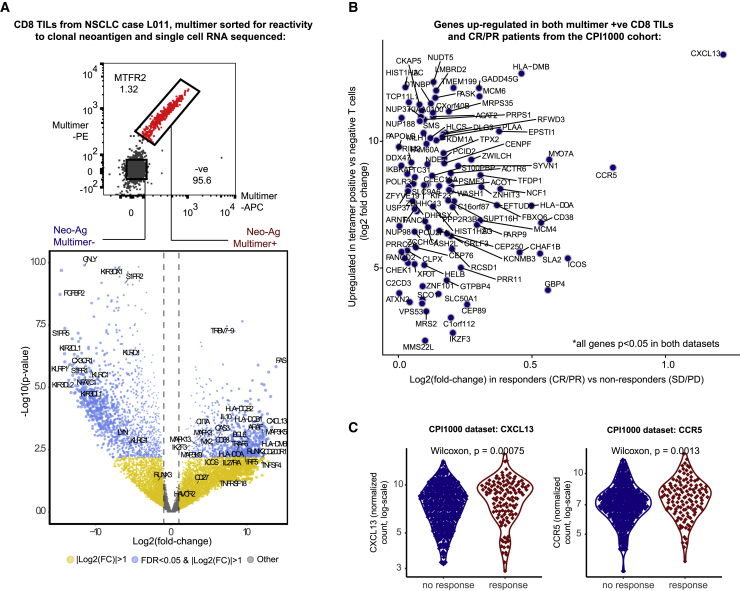
CD8^+^ neoantigen-reactive single-cell RNA-seq and CPI1000+ cohort analysis (A) Single-cell RNA sequencing (RNA-seq) data from neoantigen multimer negative versus positive CD8^+^ TILs. The top plot shows the sorting of multimer positive versus negative T cells, and the bottom plot shows differential gene expression analysis between multimer-positive versus multimer-negative cells, with log2 fold change (FC) shown on the x axis and −log10 value on the y axis. Significant genes with > 2 FC upregulation (log2(FC) > 1) and false discovery rate (FDR) < 0.05 are shown blue. (B) The same FC upregulation values from (A) on the y axis and then overlaid on the x axis is upregulation scores from the CPI1000+ cohort (log2[FC] values for responders “PR/CR” versus nonresponders “SD/PD”). The panel only shows genes significantly upregulated in both experiments. (C) Patient-level mRNA data for the two most strongly unregulated genes (*CXCL13* and *CCR5*) from (B) from the CPI1000+ cohort, with p-value derived from Mann-Whitney U test..

The identification of clonal mutation burden as the biomarker with strongest effect size in the CPI1000+ cohort implicates a central role for T cell responses targeting clonal neoantigens during immunotherapy. To examine whether genes expressed by clonal neoantigen-reactive T cells could help further elucidate the drivers of CPI response, we performed single-cell RNA sequencing (RNA-seq) on *ex vivo* CD8 TILs from a treatment-naive NSCLC patient (L011) sorted according to positivity for a clonal neoantigen (*MTFR2*) multimer (as previously described; McGranahan et al., 2016). 846 genes were significantly upregulated in multimer-positive (Mult^+^) cells relative to multimer-negative (Mult^−^) cells from the same region (q < 0.05; Figure 7A), including major histocompatibility complex class II (MHC class II) presentation machinery (e.g., *HLA-DOA* and *HLA-DMB*) and glycoprotein enzymes upregulated during T cell activation (e.g., *CD38*), trafficking (*CCR5*), and T cell dysfunction (*CXCL13*, *IL-10*, *IL27RA*, *FAS*, and *MYO7A*) (Figure 7A). Of the genes significantly enriched in Mult^+^ cells (>2-fold upregulation and q < 0.05), 101 were also significantly more highly expressed in responders (“CR/PR”) versus nonresponders (“SD/PD”) in the CPI1000+ cohort dataset (p < 0.05) (Figure 7B). *CXCL13* exhibited the most marked selective expressions in CPI responders (Figure 7B) and was the second highest differentially expressed gene in Mult^+^ cells (log2 fold change [FC] = 13.4 versus Mult^−^, q = 0.0047) (Figures 7A–7C). We note this result validates recent work from Thommen et al. (2018), and taken together, highlights that *CXCL13* expression may be a feature of clonal neoantigen-reactive CD8 TILs that associates with CPI outcome in a pan-cancer cohort. The gene next most highly expressed in responders was *CCR5*, a chemokine receptor central to T cell migration within draining lymph nodes and tumor tissues, which was also significantly higher in Mult^+^ cells (log2 FC = 8.9 versus Mult^−^, q = 0.002) (Figures 7A–7C). To control for the possibility that in the CPI1000+ patient data high *CXCL13/CCR5* expression merely reflects higher *CD8* infiltration, we tested a logistic regression model with *CD8* only compared to *CD8 + CXCL13 + CCR5* and found the latter model to be significantly better (p = 0.05, likelihood ratio test). Other notable genes significantly upregulated in Mult^+^ cells and selectively expressed by responders in the CPI1000+ cohort included co-stimulatory molecules targeted by immunotherapeutic antibodies under clinical investigation (*ICOS*), negative regulators of TCR signaling or cytokine production (*SLA2, IKZF3*), loci associated with IFN activity and predisposition to autoimmunity (*NCF1*, *EPSTI1*, and *PARP9*) or allograft rejection (*GBP4*), and regulators of type I IFN signaling (*FBX06*) (Figure 7B). These data suggest that expression of molecular circuits related to chemotaxis, T cell activation, IFN signaling, and T cell exhaustion may help to identify patients that will benefit from CPI and allude to potential immunological networks involving neoantigen reactive T cells that may confer sensitivity of tumors to immunotherapy.

## Discussion

Here, we present meta-analysis of data across >1,000 patients to assess the reproducibility of CPI response predictors across seven different tumor types. Our combined pan-cancer approach is supported by the common role for tumor antigen recognition and consequent initiation of T cell effector responses that underlie the activity of CPI in a breadth of clinical and experimental models, irrespective of tumor type (Havel et al., 2019). Although clonal TMB and TMB were strongly correlated, clonal TMB emerged as the predictor with strongest effect size and subclonal TMB being nonsignificant. In terms of markers of immune infiltration, *CXCL9* expression had the highest ranking effect size, outperforming CD8 effector and T cell inflamed signatures. In addition to subclonal TMB, a number of other putative predictors also failed to show consistent evidence of association with response. It is important to note the failure of individual markers to reach statistical significance across all seven tumor types does not rule out their importance in specific histology or drug contexts, nor does it undermine their potential biological relevance. A notable observation from this study is the relative homogeneity in CPI biomarker associations across histologies, as >80% of the biomarkers significant in individual histologies (Figure 2B) were also significant at the pan-cancer level. Indeed, histology-specific biomarkers were rare (less than five examples identified). However, we note the statistical power in individual histologies is reduced, and as sample sizes increase, additional histology-specific associations may emerge. Adding further complexity, we identified significant differences in effect size between histologies, with TMB, for example, having significantly weaker predictive utility in melanoma as compared to urothelial carcinoma.

To improve the clinical utility of biomarker stratification in immunotherapy-treated patients, progress is required in two areas: (1) the array of biomarkers identified in a research context needs to be validated and simplified into a single clinical grade test, and (2) evidence is needed to validate that sufficiently high AUC values can be attained with such a test and that this provides useful information to support clinical decision-making. In this context, we propose a multivariable model trained on >1,000 samples and validated in three independent test cohorts, which attains an AUC value of 0.86 in a pan-tumor independent test cohort, superior to TMB alone (0.68). In the short term, the most promising translational strategy is likely to be a panel or exome sequencing approach combined with a targeted gene expression quantification assay. Such a combination would allow the critical measures of antigenicity (e.g., TMB, clonal TMB, and indel TMB) and immune infiltration (e.g., *CXCL9*, *CD8A*, *CD274*, and *CXCL13*) to be captured in a cost-effective scalable manner. Regarding antigenicity, our data would support the utility of exome sequencing over panel-based approaches, given the importance of mutational signatures such as APOBEC, Tobacco, and UV. Hence, a beneficial path forward would be for a standardized assay to be established, combining exome sequencing and targeted gene expression data (e.g., NanoString) to give a multivariable predictive score of CPI response. An important question is whether predictive models will need to be customized for each cancer/treatment type or if a pan-cancer approach is sufficient. We note that with current sample sizes, the vast majority of biomarkers that were significant in one cancer type also achieved pan-cancer significance. Also, without multiple well-powered cohorts in each specific histology/drug combination, it is difficult to differentiate between study-specific phenomena and genuine tumor/drug-type-specific biology. Thus, until datasets mature in size to thousands of samples per specific combination, a pan-cancer predictive model may provide the most reliable utility. Clearly, any predictive test would require further validation in either a prospective study or additional large-scale retrospective cohorts to assess if the negative and positive predictive values can indeed exceed the threshold of clinical utility. One final observation from this study is the distinction between associations of biological/mechanistic relevance, compared to reproducible clinical grade biomarkers. Hence, caution should be applied in translating research findings into clinical practice too rapidly.

In the longer term, further discovery work is required to build a more complete understanding of CPI response, and in this context, our analysis shows that previously published biomarkers explain only ∼0.6 of the variance in CPI outcome. To address this gap, we conducted additional discovery analysis, identifying a number of other pan-cancer factors influencing CPI response, namely 9q34.3 (*TRAF2*) loss, *CCND1* amplification, DNV count, and expression of *CXCL13*. 9q34.3 (*TRAF2*) loss was found to occur via the evolutionary phenomenon of collateral sensitivity (Zhao et al., 2016), where whole-chromosome 9 loss creates a strong pro-tumor driver effect in untreated patients, which then switches to vulnerability under CPI therapy. From a clinical perspective, the observation of *CCND1* amplification as a cause of CPI resistance may offer potential therapeutic relevance, either as genetically defined subgroup unlikely to benefit from anti-PD-1/PD-L1 treatment or as a population suitable for combined CPI/anti-CDK4/6 therapy. We note the prognostic role of *CCND1* outside of immunotherapy response is complex and well studied (Watkins et al., 2020), with some reports highlighting reduced survival in *CCND1*-amplified tumors (Mahdey et al., 2011; Seiler et al., 2014; Vízkeleti et al., 2012; Wang et al., 2012), others reporting the opposite (Li et al., 2020; Ren et al., 2014), and a recent large study finding no association (Smith and Sheltzer, 2018). Analysis of mutational processes revealed a potential immunogenic role for DNVs through generation of more radical amino acid substitutions and a shift toward more hydrophobic epitopes, a known driver of immune response. Lastly, we show with single-cell RNA-seq that *CXCL13*, a marker of exhausted T cells in multiple human cancers, is preferntially expressed in both T cells reactive to a clonal neoantigen and responders in the CPI1000+ cohort. This provides independent validation of prior work by Thommen et al. (2018) and suggests that neoantigen reactivity is coupled to a *CXCL13*-secreting “exhausted” phenotype, possibly induced by chronic TCR signaling, as we have recently proposed occurs in NSCLC (Ghorani et al., 2020). If we combine the insights from published biomarker analysis with discovery results, a number of summary observations emerge. Starting within the tumor genome, we find CPI response to be underpinned by a high burden of clonal mutations enriched for immunogenic characteristics such as APOBEC or dinucleotide signatures. Armed with this rich baseline level of antigenicity, elevated *CXCL9* expression then supports ongoing recruitment of cytotoxic CD8^+^ T cells. The selective expression of *CCR5* and *CXCL13* in neoantigen-specific T cells further suggests that a key feature of CPI-responsiveness is the ability to sustain ongoing priming and recruitment of tumour reactive T cells supported by CXCR5+ lymphocytes, which may include T and B cells (Helmink et al., 2020). This model can then be perturbed by tumor-intrinsic alterations, promoting either resistance (e.g., *CCND1* amplification) or sensitization (e.g., 9q34 [*TRAF2*] loss) to treatment. While simplified in nature, these results serve to highlight recurrent features of CPI response across multiple cancer types.

Regarding study limitations, we acknowledge that the CPI1000+ cohort is made up from a diverse set of underlying previously published studies; however, the bioinformatics processing and clinical classifications have been fully harmonized. Second, we note that IHC PD-L1 data are only available in a minority of cohorts, and hence, we have estimated expression at the mRNA rather than protein level in the CPI1000+ cohort. Lastly, we note the single-tumor-region nature of the CPI1000+ dataset means that subclonal mutation counts are underestimated, impairing our ability to observe an association (or lack thereof) between subclonal mutation burden and response. In summary, here, we build and utilize a large cohort of CPI-treated patients that can be extended as new data emerge, with whole-exome sequencing and transcriptomic data, to enable a greater understanding of the determinants of treatment response. As biomarker datasets continue to grow in size, there is tangible opportunity to build a more complete understanding of CPI response, which holds the promise of augmenting immune surveillance and disease control in molecularly defined patient cohorts.

## STAR★methods

### KEY RESOURCES TABLE

**Table:** 

REAGENT or RESOURCE	SOURCE	IDENTIFIER
**Antibodies**		

Mouse anti-human CD3 BV711	Biolegend	300464; RRID: AB_2566036
Mouse anti-human CD4 AF700	Biolegend	344622; RRID: AB_2563150
Mouse anti-human CD8 BV510	Biolegend	301048; RRID: AB_2561942
Live/dead exclusion Alexa Fluor-ef780	ThermoFisher Scientific	65-0865-18
Streptavidin PE	Biolegend	405203
Streptavidin APC	Biolegend	405207

**Chemicals, peptides, and recombinant proteins**		

BD FACS Aria Fusion	Becton Dickinson	cat # 656700
C1 Single cell autoprep capture instrument	Fluidigm	N/A
EVOS FL Auto Imaging System	Thermo Fisher Scientific	N/A
Bovine serum albumin	Sigma-aldrich	9048468
phosphate buffered saline	Fisher Scientific	cat # 14190

**Critical commercial assays**		

HiSeq 2500 Sequencing System	Illumina	SY-401-2501
SMARTer v4 Ultra Low RNA Kit	Takara Clontech	634892
Qubit dsDNA HS	Thermo Fisher Scientific	Q32851
C1 Single cell IFC 10-17um diameter	Fluidigm	1006041
Nextera XT DNA Sample Preparation kit	Illumina	FC-121-1030
NextSeq 500 (150bp paired end kits)	Illumina	SY-415-1001

**Software and algorithms**		

Flowjo for MAC v10.6.2	Becton Dickinson	N/A
FacsDIVAv9.0	Becton Dickinson	N/A
Burrows-Wheeler Aligner (BWA) v0.7.15	Li and Durbin, 2009	http://bio-bwa.sourceforge.net/
Samtools v1.3.1	Li and Durbin, 2009	http://samtools.sourceforge.net/
Picard 1.81	N/A	http://broadinstitute.github.io/picard/
Mutect v1.1.7	Cibulskis et al., 2013	https://software.broadinstitute.org/cancer/cga/mutect
VarScan v2.4.1	Koboldt et al., 2012	http://varscan.sourceforge.net/
Annovar	Wang et al., 2010	http://annovar.openbioinformatics.org/en/latest/
R package ‘Copynumber’	Nilsen et al., 2012	http://bioconductor.org/packages/release/bioc/html/copynumber.html
ASCAT	Van Loo et al., 2010	https://github.com/Crick-CancerGenomics/ascat

### Resource availability

#### Lead contact

Further information and requests for resources and reagents should be directed to and will be fulfilled by the Lead Contact, Charles Swanton (Charles.Swanton@crick.ac.uk).

#### Materials availability

This study did not generate new unique reagents.

#### Data and code availability

The code used for this manuscript is available at: https://github.com/kevlitchfield1/CPI1000_paper.

### Experimental model and subject details

#### Human clinical datasets

The CPI1000+ cohort utilizes raw whole exome and RNA sequencing data from the following studies:

1. Snyder et al. (Snyder et al., 2014), an advanced melanoma anti-CTLA-4 treated cohort.
2. Van Allen et al. (Van Allen et al., 2015), an advanced melanoma anti-CTLA-4 treated cohort.
3. Hugo et al. (Hugo et al., 2016), an advanced melanoma anti-PD-1 treated cohort.
4. Riaz et al. (Riaz et al., 2017), an advanced melanoma anti-PD-1 treated cohort.
5. Cristescu et al. (Cristescu et al., 2018) an advanced melanoma anti-PD-1 treated cohort.
6. Cristescu et al. (Cristescu et al., 2018) an advanced head and neck cancer anti-PD-1 treated cohort.
7. Snyder et al. (Snyder et al., 2017), a metastatic urothelial cancer anti-PD-L1 treated cohort.
8. Mariathasan et al. (Mariathasan et al., 2018), a metastatic urothelial cancer anti-PD-L1 treated cohort.
9. McDermott et al. (McDermott et al., 2018), a metastatic renal cell carcinoma anti-PD-L1 treated cohort.
10. Rizvi et al. (Rizvi et al., 2015), a non-small cell lung cancer anti-PD-1 treated cohort.
11. Hellman et al., an unpublished cohort of non-small cell lung cancer samples treated with anti-PD-1.
12. Le et al. (Le et al., 2015), a colorectal cancer cohort treated with anti-PD-1 therapy.

In order to allow studies to be grouped by histology, additional patients from the KEYNOTE-028 and KEYNOTE-012 cohorts from Cristescu et al. were utilized to create two additional cohorts, cohort 13: Cristescu et al. urothelial cancer and cohort 14: Cristescu et al. breast cancer. For cohort 2, in line with the original authors separate categorization (Van Allen et al., 2015), the additional cohort of n = 10 patients who achieved long-term survival but with early tumor progression were excluded. For cohort 1, in line with previous treatment by (Miao et al., 2018), tumor samples from non-responding lesions from patients who otherwise had clinical benefit from immune checkpoint therapy were excluded (n = 8). Samples with truncated raw fastq file downloads, which remained truncated after multiple download attempts were also excluded. A breakdown of sample numbers for each study/histology is contained in Table S1. For validation purposes the following cohort was utilized: Cristescu et al. (Cristescu et al., 2018) “all other tumor types” (n = 76) cohort (from KEYNOTE-028 and KEYNOTE-012 studies), treated with anti-PD-1. This was reserved as a test validation cohort for the Figure 3 multivariable model analysis (i.e., not included in the Figure 2 meta-analysis), on account of its set of pan-cancer “other tumour type” mix of patients, which was selected as suitable for validation of a pan-cancer predictive model. Two additional test validation cohorts were utilized from recently published papers (Liu et al., 2019) and (Shim et al., 2020) as additional test samples for the multivariable model (Figure 3), with data being taken from supplementary tables of these papers. Cohort 9 (McDermott et al., 2018) comprised a treatment arm with anti-PD-L1 and anti-VEGF treatment, these samples were excluded from the meta-analysis of previously published biomarkers (Figure 2) and multivariable AUC analysis (Figure 3) but retained for the discovery analysis (Figure 5 and Figure 6) to maximize discovery power. Similarly, the “all other tumor types” (n = 76) set was also used in (Figure 5 and Figure 6) discovery analysis. Regarding prior lines of treatment, we note (n = 55, 5.5% of patients) had either undergone prior line of anti-CTLA-4 treatment or the biopsy was taken on treatment (see Table S1). Age and gender information of each cohort is available in the corresponding references. To assess if sex affected the results of the Figure 2 meta-analysis, the analysis was repeated including sex as an additional term in the model, and no difference was observed in the top ranked biomarkers. Validation data for copy number analysis was reused from Samstein et al. (Samstein et al., 2019), a cohort of 1662 patients treated with CPI and profiled using the MSK-IMPACT gene panel (referred to as the MSK1600 cohort). Segment copy number data for these samples was downloaded from the GENIE Synapse portal (syn7222066), https://www.synapse.org/, and clinical data were utilized from the Samstein et al. paper. In addition, a cohort of MSK-IMPACT sequenced, but non-CPI treated patients was utilized for negative control analyses, to distinguish CPI predictive from generally prognostic biomarkers. Copy number segment data for this non-CPI cohort were similarly obtained from the GENIE Synapse portal (syn7222066), https://www.synapse.org/, and clinical response data were reused from Bielski et al. (Bielski et al., 2018), and patients overlapping with the Samstein et al. were removed. Lastly, single cell RNA sequencing was conducted on CD8 TILs from patient L011, a patient diagnosed with non-small cell lung cancer who underwent definitive surgical resection prior to receiving any adjuvant therapy. Patient L011 was a 49 year old female smoker (45 pack years). Informed consent was obtained under study UCLHRTB 10/H1306/42.

### Method details

#### Clinical end points

In the CPI1000+ cohort, a uniform clinical end-point of response was defined across all the 15 studies based on radiological response as per the RECIST criteria, with “CR/PR” being classified as a responder and “SD/PD,” as well as any “NE” cases, being classed as a non-responder. We note this is a definition of response that may undercount the number of patients who derive clinical benefit, as patients with SD and extended survival have in some previous studies been considered as experiencing clinical benefit from treatment. Conversely, a radiological complete or partial response does not always equate to extended survival, and subtle distinctions between these measures should be recognized. However the “CR/PR” versus “SD/PD” definition used here allows for uniform consistency across cohorts, clearest interpretation and is consistent with the most recent literature (Cristescu et al., 2018; Mariathasan et al., 2018). For RECIST response evaluations we utilized the clinical data provided by the original authors, which in > 90% of cases was best response time point. In a minority of cases the time point of RECIST evaluation was not directly specified. For the (Cristescu et al., 2018) cohort response labels were not available as a supplementary file, however they could be inferred from cross-reference of Table S2 and Figure S3 of that paper, and validated by re-computing p values from the paper to ensure exact match (e.g., Figure 2 multivariable model p values stated in the paper, we were able to match to the 4 decimal places accuracy provided in the paper). In addition, the inferred labels were further validated when we checked the numbers of responders per detailed histology in Table S3 of (Cristescu et al., 2018) and found the inferred data matched exactly the reported results. RECIST response data was not available for the MSK1600 cohort, so instead overall survival was used as the clinical end-point, combined with negative control analysis in MSK-IMPACT profiled samples not treated with CPI, to distinguish predictive from prognostic biomarkers.

#### Multimer sorting of neoantigen reactive T cells

We have previously identified CD8+ neoantigen reactive T cells (NARTs) targeted against a clonal neoantigen (arising from the mutated *MTFR2* gene) in NSCLC tumor regions derived from patient L011 (McGranahan et al., 2016). Briefly, neoantigen-specific CD8 T cells were identified using high throughput MHC multimer screening of candidate mutant peptides generated from patient-specific neoantigens of predicted < 500nM affinity for cognate HLA as previously described (McGranahan et al., 2016). 288 candidate mutant peptides (with predicted HLA binding affinity < 500nM, including multiple potential peptide variations from the same missense mutation) were synthesized and used to screen expanded L011 TILs. In patient L011, TILs were found to recognize the HLA-B^∗^3501 restricted, MTFR2D326Y-derived mutated sequence FAFQEYDSF (netMHC binding score: 22nM), but not the wild-type sequence FAFQEDDSF (netMHC binding score: 10nM). No responses were found against overlapping peptides AFQEYDSFEK and KFAFQEYDSF. Neoantigen-specific CD8+ T cells were tracked with peptide-MHC multimers conjugated with either streptavidin PE (Biolegend, cat#405203), APC (Biolegend, cat#405207) BV650 (Biolegend, cat#405231) or PE-Cy-7 (Biolegend, cat#405206) and gated as double positive cells among live, single CD8+ cells. Phenotypic characterization of neoantigen-specific CD8 T cells in L011 was performed as previously described (McGranahan et al., 2016).

#### Single-cell RNA sequencing of neoantigen reactive T cells

Multimer-positive and negative single CD8+ T cells from NSCLC specimens were sorted directly into the C1 Integrated Fluidic Circuit (IFC; Fluidigm). Cell lysing, reverse transcription, and cDNA amplification were performed as specified by the manufacturer. Briefly, 1000 single, multimer positive or negative CD8 T cells were flow sorted directly into a 10- to 17-μm-diameter C1 Integrated Fluidic Circuit (IFC; Fluidigm). Ahead of sorting, the cell inlet well was preloaded with 3.5 μL of PBS 0.5% BSA. Post-sorting the total well volume was measured and brought to 5 μL with PBS 0.5% BSA. 1 μL of C1 Cell Suspension Reagent (Fluidigm) was added and the final solution was mixed by pipetting. Each C1 IFC capture site was carefully examined under an EVOS FL Auto Imaging System (Thermo Fisher Scientific) in bright field, for empty wells and cell doublets. An automated scan of all capture sites was also obtained for reference. Cell lysing, reverse transcription, and cDNA amplification were performed on the C1 Single-Cell Auto Prep IFC, as specified by the manufacturer. The SMARTer v4 Ultra Low RNA Kit (Takara Clontech) was used for cDNA synthesis from the single cells. cDNA was quantified with Qubit dsDNA HS (Molecular Probes) and checked on an Agilent Bioanalyzer high sensitivity DNA chip. Illumina NGS libraries were constructed with Nextera XT DNA Sample Preparation kit (Illumina), according to the Fluidigm Single-Cell cDNA Libraries for mRNA sequencing protocol. Sequencing was performed on Illumina® NextSeq 500 using 150bp paired end kits.

### Quantification and statistical analysis

#### Sample quality control

**Figure S5:**
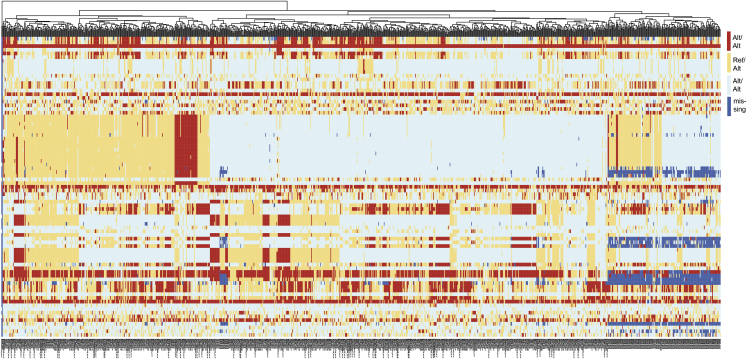
Clustering by common germline SNP panel to ensure no duplicate participants were recorded in the CPI1000+ cohort, related to STAR methods Columns are patients, rows are SNPs.

**Figure S6:**
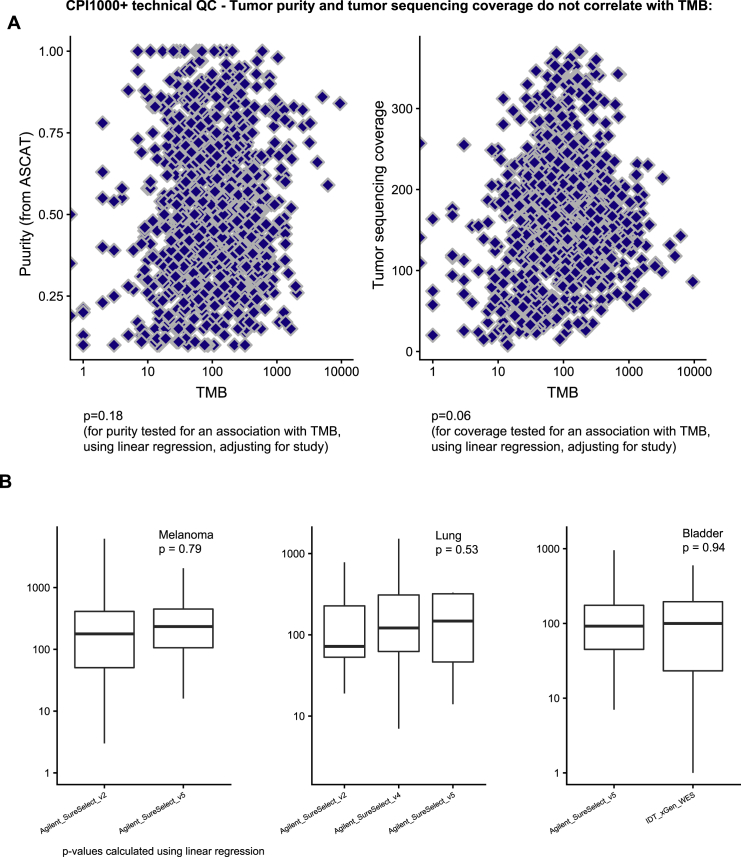
Purity, sequencing coverage, and choice of exome capture kits do not correlate with TMB scores in the CPI1000+ cohort, related to STAR methods

**Figure S7:**
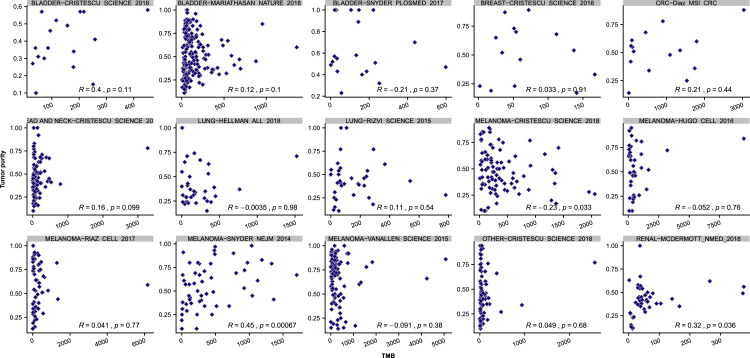
Purity vs TMB correlations by study, related to STAR methods

First, samples were clustered using a panel of common germline SNPs, to ensure no duplicate participants were included (Figure S5). Next, we assessed for any technical correlations between mutation counts and purity or sequencing coverage (Figure S6A). While at the combined CPI1000+ cohort level we did not observe any significant relationships (Figure S6A), we note in a minority of individual studies (3 out of 15) there was a significant relationship between TMB and purity (Figure S7). This technical correlation is linked to low sequencing coverage, for example the Snyder et al. NEJM 2014 cohort has the strongest correlation and is also the cohort with lowest average depth per tumor sample. These findings are consistent with recent results published by Anagnostou et al. (Anagnostou et al., 2020), who demonstrate a relationship between purity and TMB, which is mitigated with higher coverage. Finally, we assessed for any evidence of different exome capture kits across the cohorts impacting results, and found no significant difference in TMB scores based on exome capture kits utilized (Figure S6B). We note however that Agilent SureSelect kits were used in nearly all studies, except for one cohort, Snyder et al. (Snyder et al., 2017), which used IDT xGen WES capture, and in addition we found no specification of the capture kit used in the Hugo et al. manuscript (Hugo et al., 2016).

#### Whole exome sequencing (DNA) pipeline–variant calling

For all studies we obtained germline/tumor whole exome sequencing data in either BAM, SRA or FASTQ format, from the relevant sequencing repository or directly from the original authors, and where applicable reverted these files back to FASTQ format using Picard tools (version 1.107) SamToFastq. Raw paired-end reads in FASTQ format were aligned to hg19 obtained from the GATK bundle (v2.8) using bwa mem (bwa v0.7.15) (Li and Durbin, 2009; McKenna et al., 2010). Picard tools (picard v1.107) was used to remove duplicates (http://broadinstitute.github.io/picard), and GATK was additionally used for local indel realignment. Quality control metrics were produced with picard tools (v1.107), FastQC (v0.11.5 - http://www.bioinformatics.babraham.ac.uk/projects/fastqc/) and GATK(v3.9). Platypus v0.8.1 was used to call homozygous and heterozygous germline SNPs (Rimmer et al., 2014). The default parameters were used, but the genIndels flag was set to FALSE. Only SNPs with a minimum depth of coverage of 20x were taken forward. Somatic variants were detected using two tools (MuTect v1.1.7 & VarScan2 v2.4.1) (Cibulskis et al., 2013; Koboldt et al., 2012), using the following method: SAMtools mpileup (version 0.1.19) was used to locate non-reference positions in tumor and germline samples. Bases with a Phred score of less than 20 or reads with a mapping quality less than 20 were omitted. The Base alignment quality (BAQ) calculation option was deactivated and a threshold of 50 was set for the coefficient of downgrading mapping quality. VarScan2 somatic (version 2.3.6) used output from SAMtools mpileup to identify somatic variants between tumor and matched germline samples. VarScan2 processSomatic was used to extract the somatic variants. Single nucleotide variant (SNV) calls were filtered for false positives with the associated fpfilter.pl script in Varscan2, initially with default settings then repeated with min-var-frac = 0·02, having first run the data through bam-readcount (version 0.5.1). MuTect (version 1.1.4) was also used to detect SNVs, and results were filtered according to the filter parameter PASS. Default parameters were used in both tools with the exception of: i) minimum coverage for the germline sample was set to 10, ii) minimum coverage for the tumor sample was set to 30 and minimum alternative allele read depth of 3, iii) minimum somatic variant allele frequency (VAF) was set to 0.01 and minimum alternative read coverage set to 5, iv) alternative reads in the germline had to be ≤ 5 and germline VAF ≤ 1%, v) variant had to be not present in EXAC03 database at 5% or higher frequency. In final QC filtering, an SNV was considered a true positive if the variant allele frequency (VAF) was greater than 1% and the mutation was called by both VarScan2, with a somatic p value ≤ 0.01, and MuTect. Alternatively, a frequency of 5% was required if only called in VarScan2, again with a somatic p value ≤ 0.01. For small scale insertion/deletions (INDELs), the same filters above were applied, and only calls classified as high confidence by VarScan2 processSomatic were kept for further analysis, with somatic_p_value scores less than 1.0 × 10^−^^3^. Variant annotation was performed using ANNOVAR (version 2016Feb01) (Wang et al., 2010).

#### Whole exome sequencing (DNA) pipeline–copy number calling

VarScan2(v2.4.1) was used to generate logR depth ratios from paired tumor region/germline samples. These values were subsequently GC corrected (Cheng et al., 2011). Default parameters were used to generate this data with the exception of: min-coverage = 8 and min-segment-size = 50. B-Allele Frequencies (BAFs)–the proportion of reads with a SNP variant relative to the total read depth–were calculated using the SNPs called in the germline by platypus. The GC-corrected logR values and BAF values are then used by ASCAT (v2.3) (Van Loo et al., 2010) to generate segmented allele-specific copy number data, including estimates of tumor ploidy and cellularity. Sequenza (Favero et al., 2015) was additionally run on all samples in parallel. To ensure accuracy, default ASCAT copy number solutions were quality control checked, and where a sample failed any of the following quality flags it then underwent manual review: i) unexpectedly high purity, defined as tumor cellularity > 80%, ii) unexpectedly low levels of loss of heterozygosity, defined as fraction of the genome LOH of < 0.1, iii) unexpectedly high level of the genome with both alleles at even copy number, defined as the fraction of the genome with alleles A and B both even as > 0.7, iv) unexpectedly high level of the genome with copy number = 0, defined as ≥ 4Mb with copy number = 0. In addition, an orthogonal measure of tumor purity was derived based on mutation variant allele fraction, as previously described (Jamal-Hanjani et al., 2017), and samples with a mismatch in purity between ASCAT and orthogonal measurements of greater than 1 standard deviation were additionally flagged for manual review. Samples that had been flagged for manual review underwent dual analyst inspection, which involved review of the default and alternative copy number solutions from ASCAT and Sequenza tools. Where a better fitting solution was available (based on the rules above, as well as obtaining consistency in solutions between ASCAT and Sequenza) this was utilized rather than the ASCAT default.

#### RNA sequencing pipeline

RNAseq data was obtained in BAM/SRA/FASTQ format for all studies, and reverted back to FASTQ format using bam2fastq (v1.1.0). FASTQ data underwent quality control and were aligned to the hg19 genome using STAR (Dobin et al., 2013). Expression counts were normalized using DESeq2 variance stabilizing transformation (vst) function and transcripts per kilobase million (TPM) values calculated using RSEM with default parameters (Li and Dewey, 2011). Within the meta-analysis and multivariable modelling sections (Figures 2 and 3), individual gene expression analyses across samples were conducted using vst normalized expression counts, and for signatures involving multiple genes, TPM values were used (to give consistency with the majority of original authors methodologies). For other RNAseq analyses the relevant measure used is indicated in the axis labels.

#### Mutation clonality analysis

PyClone (Roth et al., 2014) version 0.12.7 was used to determine the clonal status of mutations. For each sample variant calls were integrated with local allele specific copy number (obtained from ASCAT), tumor purity (also obtained from ASCAT), and variant allele frequency data. All mutations were then clustered using the PyClone Dirichlet process clustering. This enables mutations to be grouped together based on likely co-occurrence in the same set of cancer cells (clones), from which the founding (truncal) clone can be identified. We ran PyClone with 10,000 iterations and a burn-in of 1000, and using parameters as previously described (Jamal-Hanjani et al., 2017).

#### HLA and neoantigen analysis

Neoantigen predictions were derived by first determining the 4-digit HLA type for each patient, along with mutations in class I HLA genes, using POLYSOLVER (Shukla et al., 2015). Next, all possible 9, 10 and 11-mer mutant peptides were computed, based on the detected somatic non-synonymous SNV and INDEL mutations in each sample. Binding affinities of mutant and corresponding wild-type peptides, relevant to the corresponding POLYSOLVER-inferred HLA alleles, were predicted using NetMHCpan (v3.0) and NetMHC (v4.0) (Andreatta and Nielsen, 2016). Neoantigen binders were defined as IC_50_0 < 500 nM or rank < 2.0. Grantham distances between HLA gene allele pairs were calculated using the same procedure described in Pierini et al. (Pierini and Lenz, 2018), utilizing the Grantham distance metric originally designed for investigating protein evolution from physiochemical differences in amino acid sequences (Grantham, 1974). Aligned protein sequences for HLA alleles were obtained from the IMGT database (Robinson et al., 2016) for the different HLA alleles as called by Polysolver from the raw germline data files for the HLA-A, B and C genes. A custom R script was created to calculate the Grantham distance at each position on exons 2 and 3 of two aligned HLA alleles (exon 2 and 3 being the peptide binding region of the HLA protein). The final Grantham distance score between two HLA alleles was calculated as the sum of the scores at each position divided by length of the amino acid sequence. The average Grantham score for an individual patient was then calculated by taking the mean of the separate Grantham scores for HLA-A, B and C. It should be noted that to be consistent with the approach used in Pierini et al. (Pierini and Lenz, 2018), these scores do not correct for possible loss of heterozygosity of the HLA alleles (LOHHLA) that frequently occur somatically during cancer evolution but instead reflect the germline HLA divergence of a patient pre-cancers. HLA loss of heterozygosity analysis was performed using the LOHHLA tool as previously described (McGranahan et al., 2017). We implemented two additional filters to the HLA LOH calls. The first is based on the expected depth of the HLA allele in the tumor. This is calculated as the depth of the allele in the germline divided by the tumor purity and multiplied by the ratio of the number of unique reads in the tumor to the germline bam, where the allele depth in the germline sample is the median depth across the mismatches. We filtered out calls for genes that had at least one allele with an expected depth in the tumor of less than 10. We also filtered out HLA LOH calls for genes that had a minor copy number less than −0.5.

#### Literature search

PubMed abstract/title fields were searched for the following sets of keywords:

“Predictive biomarker AND immunotherapy,” “Associated AND checkpoint inhibitor response,” “Sensitivity AND PD-1 blockade,” “Sensitivity AND CTLA-4 blockade,” “Sensitivity AND PD-L1 blockade,” “Resistance AND PD-1 blockade,” “Resistance AND CTLA-4 blockade,” “Resistance and PD-L1 blockade,” “Immunotherapeutic AND escape mechanism,” “Predictors AND immune checkpoint blockade,” “Immune checkpoint blockade AND determinants,” “Immune checkpoint blockade AND markers,” “Cancer immunotherapy AND determinant,” “Effectiveness AND immune checkpoint inhibitors,” “Prediction AND immune checkpoint blockade,” “Predict AND cancer immunotherapy,” “Predictive biomarkers AND checkpoint blockade therapies,” “Response AND checkpoint blockade immunotherapy,” “Predicts AND checkpoint blockade immunotherapies,” “Prediction AND immunotherapies.” Articles matching human species and date range [2015-2020] were selected, yielding a total of 723 unique manuscripts. The final search was conducted on 12^th^ August 2020. Each paper was reviewed, and where human data was presented to support a biomarker associated with checkpoint inhibitor response, this was added to the analysis. Case reports were excluded, along with review papers, and biomarkers that could not be calculated with exome or transcriptome data. In total 55 unique biomarkers were identified and included in the study for analysis.

#### Derivation of published biomarkers

The following previously published biomarkers were tested for association with response to CPI therapy: tumor mutation burden (TMB) (Rizvi et al., 2015; Snyder et al., 2014; Van Allen et al., 2015) (also split out into Clonal (McGranahan et al., 2016) and Subclonal TMB), frameshift insertion/deletion (indel) mutation burden (Turajlic et al., 2017), burden of indels escaping nonsense mediated decay (Lindeboom et al., 2019), Tobacco mutation signature (Anagnostou et al., 2020), UV signature (Knepper et al., 2019), APOBEC signature (Chapuy et al., 2019), Differential Agretopicity Index (Ghorani et al., 2018), *MUC16* neoantigens (Balachandran et al., 2017), Neoantigen fitness model (Łuksza et al., 2017), *SERPINB3/SERPINB4* mutations (Riaz et al., 2016), DNA damage response pathway mutations (Conway et al., 2018), Shannon diversity index for intratumor heterogeneity (SDI-ITH) (Wolf et al., 2019), burden of somatic copy number alterations (Davoli et al., 2017), burden of somatic copy number losses (Roh et al., 2017), HLA-I evolutionary divergence (Chowell et al., 2019), maximal HLA heterozygosity, HLA B44/B62 supertypes, HLA B1501 type (Chowell et al., 2018), *KIR3DS1* germline variants (Trefny et al., 2019), loss of heterozygosity at the HLA locus (McGranahan et al., 2017), sex (Conforti et al., 2018), *B2M* mutations (Gettinger et al., 2017), JAK1/JAK2 mutations (Shin et al., 2017), *KRAS* and *TP53* mutations (Aredo et al., 2019), *PTEN* mutations (Peng et al., 2016), *RTK* mutations (Anagnostou et al., 2020), *STK11* mutations (Aredo et al., 2019), *BAP1* mutations (Shrestha et al., 2019), *CD8A* (Tumeh et al., 2014), *CD274 (*PD-L1) (Gibney et al., 2016), *CD38* (Chen et al., 2018), *HAVCR2* (TIM3)/*LGALS9* (Koyama et al., 2016), *MEX3B* (Huang et al., 2018) and *CXCL9* expression (Chow et al., 2019), as well as the CD8 T cell effector (McDermott et al., 2018), proliferation (Pabla et al., 2019), cytolytic (Rooney et al., 2015), stroma-EMT (Wang et al., 2018), TGF beta pan fibroblast (Mariathasan et al., 2018), IMPRES (Auslander et al., 2018), CD8 T effector from the POPLAR trial (Fehrenbacher et al., 2016), 12-cheomokine (Messina et al., 2012; Tokunaga et al., 2020), HERV-3 family expression (Panda et al., 2018) and T cell inflamed gene expression signatures (Ayers et al., 2017). TMB was measured on a per megabase basis using the Friends of Cancer Research TMB Harmonization Project phase I guidelines (Merino et al., 2020), clonal TMB was measured as per (McGranahan et al., 2016) with samples which failed pyclone clustering assumed that all mutations were clonal, SCNA load was defined using the weighted genome instability index (wGII) (Endesfelder et al., 2014), expression of individual genes was measured using varianceStabilizingTransformation (vst) normalized expression count from DESeq2 (for datasets with RNaseq) or normalized nanostring expression values for the Cristescu et al. cohort. In the Cristescu et al. cohort, where transcriptome data is only available for a subset of genes, gene expression signatures were calculated with as many genes as were available. For inactivating pathway mutations (i.e., *B2M*, *PTEN, JAK1/JAK2*, DNA damage response) loss of function mutations (i.e., those causing a premature stop codon) and homozygous deletions were included. DNA damage response pathway genes were defined as: *BRCA1, BRCA2, ATM, POLE, ERCC2, FANCA, MSH2, MLH1, POLD1* and *MSH6* based on (Conway et al., 2018). All other biomarkers were defined as per the method outlined in the original underlying publication as referenced above. Associations with response were tested using logistic regression. To allow biomarkers with varying measurement scales (e.g., mutation counts versus gene expression values) to be compared equivalently based on effect size rather than p value (Wasserstein et al., 2019), all biomarker values (continuous and binary) were converted to standard z-scores (i.e., mean normalized to equal zero, and standard deviation normalized to one). To avoid data pooling, each biomarker was tested individually in each sub-study, and then the effect sizes and standard errors were combined through random effects meta-analysis to derive a final p value per biomarker. Meta-analysis was conducted using R package ‘meta’. Proportion of variance explained analysis. The total proportion of variance explained by all biomarkers was calculated by logistic regression pseudo-*R*^2^, using R function ‘PseudoR2′.

#### Fitting a multivariable model of CPI response

The predicative utility of a multivariable model was benchmarked against TMB as a univariable comparator. The multivariable model was made up of all biomarkers attaining significance in the Figure 2A meta-analysis (final column, p-meta validation cohorts only), comprising 11 measures in total: Clonal TMB, Indel TMB, NMD-escape TMB, UV signature, Tobacco signature, APOBEC signature, sex, T cell inflamed GEP signature, and gene expression values for CD274 (PD-L1), CD8A and CXCL9. All 11 biomarkers were inputted into the gradient boosted tree algorithm XGBoost (R package ‘xgboost’), a widely used machine learning algorithm effective for classification tasks. The variation in feature importance scores across tumor types was demonstrated using the largest cohort of matched exome and transcriptome data for each tumor type: urothelial: (Mariathasan et al., 2018), head and neck: (Cristescu et al., 2018), melanoma: (Cristescu et al., 2018) and renal: (McDermott et al., 2018). All training samples (n = 1008) were then utilized to build a final predictive model with the 11 biomarkers, with maximum tree depth of 2, nrounds set as 15 and eta set 0.2—these values were derived using grid search in ‘caret’ R package with 5-fold cross validation using (n = 1008) training cohort samples. All other parameters were kept as default values. TMB predictions were made using an identical model desgin. R package ‘ROCR’ was used for the ROC curve analysis. Three cohorts were utilized as independent test/validation sets (not used in model training process): 1) the KEYNOTE-028 “other tumour type” cohort from (Cristescu et al., 2018), 2) Liu, Schilling et al., 2019 melanoma cohort (Liu et al., 2019), and 3) Shim et al., 2020 lung cancer cohort (Shim et al., 2020). Test set 1) was selected as this consists of “other tumour type” samples, and the final model from Figure 3b/c is trained on a combined set of pan-cancer samples, hence this mixed tumor type cohort was selected as an appropriate validation set. Test sets 2) and 3) were selected as test datasets based on their publication timing, i.e., they are the most recently published datasets, which became available after model training was completed. Data for test cohort 1) was obtained as raw data, and data for test cohorts 2) & 3) was obtained from supplementary published tables on account of the recent publication of these studies. We note that for test cohort 3) (Shim et al., 2020) only TMB, PD-L1, smoking signature (inferred from smoking history) and sex data was available, and hence only these four variables were used in the multivariable model, and PD-L1 was used in place of *CXCL9* expression in the two-parameter model.

#### Mutation signature analysis

DeconstructSigs (Rosenthal et al., 2016) was used to derive COSMIC mutational signatures (v2) (Alexandrov et al., 2015) for each tumor samples with ≥ 50 somatic mutations (n = 872 patients). Grantham distance, which considers three properties: composition, polarity and molecular volume, was used to measure difference in amino acid properties (Grantham, 1974). A Grantham distance change of ≥ 100 was considered radical, or less than 100 conservative (Dagan et al., 2002). Hydrophobicity scores per amino acid were derived using the scale from Kyte & Doolittle (Kyte and Doolittle, 1982). Data from melanoma/gastric cancer patient tumor infiltrating lymphocyte (TIL) samples, which were screened for reactivity against neoantigen peptides, was taken from (Chudley et al., 2014; Gros et al., 2016; Tran et al., 2015).

#### Pan-cancer analysis of copy number losses and gains

Copy number segment data from ASCAT for all responders and non-responders were inputted to the R package ‘copynumber’ (Nilsen et al., 2012) to derive the gain and loss frequency across the genome for each group (i.e., for responders and non-responders separately). Region level cytoband coordinates were obtained from the UCSC Table Browser, with 286 autosomal chromosomes cytobands defined. For gains and losses (separately) the frequency per cytoband was converted back to absolute patient counts and the difference between responders and non-responders was compared using a 2x2 Fisher’s exact test. Results were corrected for multiple testing using the p.adjust function in R, with the FDR method. The frequency of whole chromosomal losses was analyzed using genome-wide SNP6 segmented data per sample from the TCGA GDAC Firehose repository (http://firebrowse.org/), for histology types overlapping with the CPI1000+ cohort, i.e., TCGA cohorts: BLCA, BRCA, COADREAD, HNSC, KIRC, LUAD, LUSC and SKCM. The immune evasion alteration analysis (Figure S3B) was conducted as per previously published method by Rosenthal et al., 2019 (Rosenthal et al., 2019), which defines antigen-presentation-pathway genes as components of the HLA enhanceosome, peptide generation, chaperones or the MHC complex itself. In the analysis we included disruptive events (non-synonymous mutations or copy-number loss defined relative to ploidy) of the following genes: *CIITA, IRF1, PSME1, PSME2, PSME3, ERAP1, ERAP2, HSPA, HSPC, TAP1, TAP2, TAPBP, CALR, CNX, PDIA3* and *B2M*. The analysis was also repeated for non-synonymous mutations only (i.e., no copy number loss events). In addition, a multivariable logistic regression test was also performed, adjusting for wGII and cancer type, which also confirmed a significant association between 9q34 loss and a higher rate of immune evasion.

#### Pan-cancer analysis of focal amplifications and deep deletions

Copy number segment data from ASCAT for all responders and non-responders were utilized to identify tumors with either focal amplification (copy number ≥ 5 and segment length < 3Mb) or homozygous deletions (copy number = 0 and segment length < 3Mb), in known oncogenes (for amplifications) or tumor suppressor genes (for deep deletions). Oncogenes and tumor suppressor genes were defined according to the Cancer Gene Census (https://cancer.sanger.ac.uk/census), accessed 23^rd^ October 2019, and events with greater than 5% frequency in the CPI1000+ cohort were analyzed. The difference in Oncogene/TSG amplification/deletion frequency was compared between responders and non-responders using a one-sided 2x2 Fisher’s exact test (events were hypothesized to associate with resistance only, as they are not collateral passenger events that may cause sensitization).

#### Analysis of single cell RNA sequencing data

All sequencing data was assessed to detect sequencing failures using FASTQC and lower quality reads were filtered or trimmed using TrimGalore. Outlier samples containing low sequencing coverage or high duplication rates were discarded. Analyses using the RNaseq data were performed in the R statistical computing framework, version 3.5 using packages from BioConductor version 3.7. The single cell RNAseq samples were mapped to the GRCh38 reference human genome, as included in Ensembl version 84, using the STAR algorithm and transcript and gene abundance were estimated using the RSEM algorithm. After quantification, the scater package was used to set filtering thresholds, based on using spike ins and mitochondrial genes to filter out bad quality cells, filtering by total number of genes and filtering by total number of sequenced reads. The remaining cells were used after normalizing using size-factors estimated by the SCRAN package. Downstream analyses used log2 transformed normalized count data. All count data, metadata and intermediate results were kept within a SummarizedExperiment/SingleCellExperiment R object. The data was processed using the edgeR BioConductor package that was used for outlier detection and differential gene expression analyses. Differentially expressed genes were assessed based on their protein coding status. The combined single cell and CPI1000+ bulk sequencing analysis was conducted as follows: i) genes discovered in single cell sequencing dataset were filtered for q < 0.05 (FDR corrected p value), log10 fold-change > 2 and T cell receptor variable genes (e.g., *TRAV19*) were removed, ii) filtering from the previous step yielded n = 846 genes, which were then each validated for an association with response in patients from the CPI1000+ cohort with full RNAseq data (n = 564). TPM expression values were used and tested for an association with response using logistic regression, with all samples combined together but corrected for study as a covariate, iii) the previous step yielded 110 genes with p < 0.05, we note these p values were not corrected for multiple testing as this was a validation of the single cell identified hits. Of the 110 genes, 101 were upregulated in CPI responders, and this was utilized for figure plotting.

#### Statistical methods

Unless otherwise stated (e.g., the section above “Derivation of published biomarkers”), odds ratios were calculated using Fisher’s exact test for count data, Kruskal-Wallis test was used to test for a difference in distribution between three or more independent groups, and Mann Whitney U test was used to assess for a difference in distributions between two population groups. Logistic regression was used to assess multiple variables jointly for independent association with binary outcomes. Overall survival analysis was conducted using a Cox proportional hazards model. Statistical analysis were carried out using R3.4.4 (http://www.r-project.org/) or greater. We considered a p value of 0.05 as being statistically significant. Any discovery analysis with more than 20 comparisons was subject to multiple testing correction using the R p.adjust function, with FDR method.

## Supplemental information

Document S1. Clinical and sequencing assay data on the CPI1000+ cohort

Table S2. Simplified two-paramter multivariable model, related to Figure 3
