# Supplementary material for: Meta-analysis of tumor- and T cell-intrinsic mechanisms of sensitization to checkpoint inhibition
Source: Cell. 2021 Feb 4;184(3):596–614.e14. doi: 10.1016/j.cell.2021.01.002 (PMC7933824; doi:10.1016/j.cell.2021.01.002)
Supplement: Document S1. Clinical and sequencing assay data on the CPI1000+ cohort [file mmc1.pdf]

Cell, Volume *184*

Supplemental Information

Meta-analysis of tumor-

and T cell-intrinsic mechanisms

# of sensitization to checkpoint inhibition

Kevin Litchfield, James L. Reading, Clare Puttick, Krupa Thakkar, Chris Abbosh, Robert Bentham, Thomas B.K. Watkins, Rachel Rosenthal, Dhruva Biswas, Andrew Rowan, Emilia Lim, Maise Al Bakir, Virginia Turati, José Afonso Guerra-Assunção, Lucia Conde, Andrew J.S. Furness, Sunil Kumar Saini, Sine R. Hadrup, Javier Herrero, Se-Hoon Lee, Peter Van Loo, Tariq Enver, James Larkin, Matthew D. Hellmann, Samra Turajlic, Sergio A. Quezada, Nicholas McGranahan, and Charles Swanton

**AUC values by cohort by predictor:**	**TMB**	**multivariable (11 markers)**	**multivariable (2 markers: clonal TMB + CXCL9)**
Test_cohort_1 - "all other" tumour types	0.68	0.86	0.79
Test_cohort_2 – melanoma	0.58	0.66	0.63
Test_cohort_3 - lung cancer	0.62	0.70	0.716

Table S2 – AUC test results for the TMB, full multivariable model, and two parameter models. Related to figure 3.
